# A paradox for air pollution controlling in China revealed by “APEC Blue” and “Parade Blue”

**DOI:** 10.1038/srep34408

**Published:** 2016-09-29

**Authors:** Haoran Liu, Cheng Liu, Zhouqing Xie, Ying Li, Xin Huang, Shanshan Wang, Jin Xu, Pinhua Xie

**Affiliations:** 1 School of Earth and Space Sciences, University of Science and Technology of China, Hefei, 230026, China; 2 Key Lab of Environmental Optics & Technology, Anhui Institute of Optics and Fine Mechanics, Chinese Academy of Sciences, Hefei 230031, China; 3 Center for Excellence in Urban Atmospheric Environment, Institute of Urban Environment, Chinese Academy of Sciences, Xiamen 361021, China; 4 Department of Ocean Science and Engineering, Southern University of Science and Technology, Shenzhen, 518055, China; 5 Institute for Climate and Global Change Research & School of Atmospheric Sciences, Nanjing University, Nanjing, 210023, China; 6 Collaborative Innovation Center of Climate Change, Jiangsu Province, China; 7 Department of Atmospheric Chemistry and Climate, Institute of Physical Chemistry Rocasolano, CSIC, Madrid, 28006, Spain

## Abstract

A series of strict emission control measures were implemented in Beijing and surrounding regions to ensure good air quality during the 2014 Asia-Pacific Economic Cooperation (APEC) summit and 2015 Grand Military Parade (Parade), which led to blue sky days during these two events commonly referred to as “APEC Blue” and “Parade Blue”. Here we calculated Multi-Axis Differential Optical Absorption Spectroscopy (MAX-DOAS) and Ozone Monitoring Instrument (OMI) NO_2_ and HCHO results based on well known DOAS trace gas fitting algorithm and WRF-Chem model (with measured climatology parameter and newest emission inventor) simulated trace gases profiles. We found the NO_2_ columns abruptly decreased both Parade (43%) and APEC (21%) compared with the periods before these two events. The back-trajectory cluster analysis and the potential source contribution function (PSCF) proved regional transport from southern peripheral cities plays a key role in pollutants observed at Beijing. The diminishing transport contribution from southern air mass during Parade manifests the real effect of emission control measures on NO_2_ pollution. Based on the ratios of HCHO over NO_2_ we found there were not only limited the NO_2_ pollutant but also suppress the O_3_ contaminant during Parade, while O_3_ increased during the APEC.

Atmospheric pollution has become a serious menace to public health all over the world, especially in China^1^,^2^. Beijing, the capital city of China, has been suffered the most gravely air pollution problems in the country^3^. In recent years, many significant events had been held in Beijing, especially for 2014 Asia-Pacific Economic Cooperation (APEC) summit and the 2015 Grand Military Parade (hereinafter called Parade). The APEC summit and Parade were regarded as the excellent examples that a series of strict emission control measures, for example curbing or halting production from power plants and factories with high emissions, limitation of vehicles and even stop all construction activities, were implemented in Beijing and surrounding regions during the APEC (Nov. 3–12) and the Parade (Aug. 20-Sep. 3)^4^,^5^. From the previous studies, these measures led to large reductions in secondary inorganic aerosol (SIA), in second organic aerosol (SOA), aerosol optical depth (AOD) and absorption aerosol optical depth (AAOD) in the APEC period, which might be the primary reason led to blue sky days during APEC commonly referred to as “APEC Blue”^4^,^5^. Similar blue sky days returned again during Parade, which was also called as “Parade Blue”. However, the O_3_, volatile organic compounds (VOCs) and NO_2_ had been barely taken into account in these researches for evaluating the contamination status during these two periods.

OMI as a commonly used satellite measurement could provide a particularly important spatial information on the concentration and distribution of the atmospheric trace gases^6^,^7^,^8^,^9^. Ground-based MAX-DOAS method has been extensively used for trace gas and aerosol measurements in the past decade^10^,^11^,^12^,^13^,^14^,^15^, which are frequently used to validate satellite observation^16^,^17^,^18^. Compared with satellite measurements, the MAX-DOAS measurements have the advantages that they can offer crucial information about daily variation of tropospheric NO_2_ and the vertical distributions over the research sites, which cannot be acquired from the satellite observations^18^. Both the results of MAX-DOAS and satellites could provide the comparable vertical column densities (VCDs)^19^. Therefore, it’s useful to compare and combine these two datasets to investigate more information.

NO_2_ usually serves as a proper indicator for the intensity of the anthropogenic emissions, due to its lifetime is not so long^5^. To explicitly probe the impact of emission and transmission perturbation on altering the air quality, we propose to use the MAX-DOAS and OMI satellite observations to retrieve the NO_2_ VCDs, and combine PSCF method to analyze the NO_2_ potential source at Beijing urban area (Fig. S1, 40°N, 116°22′48″E) during Parade and APEC periods in this study.

The previous study had demonstrated that HCHO and NO_2_ from the OMI serve as appropriate proxies for *in situ* observations of total reactive nitrogen (NO_y_) and VOC in polluted environments, just like Beijing, and their ratio is an appropriate indicator to examine the chemical sensitivity of local ozone production (PO_3_)^20^,^21^,^22^. And they found that OMI tropospheric HCHO/NO_2_ Ratio (hereafter refer to as “Ratio”) <1 represents PO_3_ reduces with diminishing in VOCs (VOC-limited conditions), and Ratio >2 represents NO_x_-limited conditions. When ratio is between 1 and 2 indicates that a transition regime (mixed VOC-NO_x_-limited regime) where the instantaneous PO_3_ can be changed by both VOC and NO_x_ emissions^21^.

In this study, we also employ the OMI products (NO_2_ and HCHO) and combine the corresponding Ratio to analyze the O_3_ variations during both Parade and APEC periods.

## Results

In order to evaluate the impact of emission control policy on air quality during Parade and APEC, three episodes are separately defined in this study: 1^st^ episodes is defined as the period of Parade (from August 20^th^ to September 3^rd^ 2015), in which the strict air quality policies were implemented at a regional scale; 2^nd^ and 3^rd^ episodes are defined as the “pre-Parade” from August 5^th^ to 19^th^ and the “post-Parade” from September 4^th^ to 21^st^. Similarly, the three episodes of APEC are respectively defined as pre-APEC period (from October 24^th^ to November 2^nd^), APEC period (from November 3^rd^ to 12^th^) and post-APEC period (from November 13^th^ to 21^st^).

**Figure 1:**
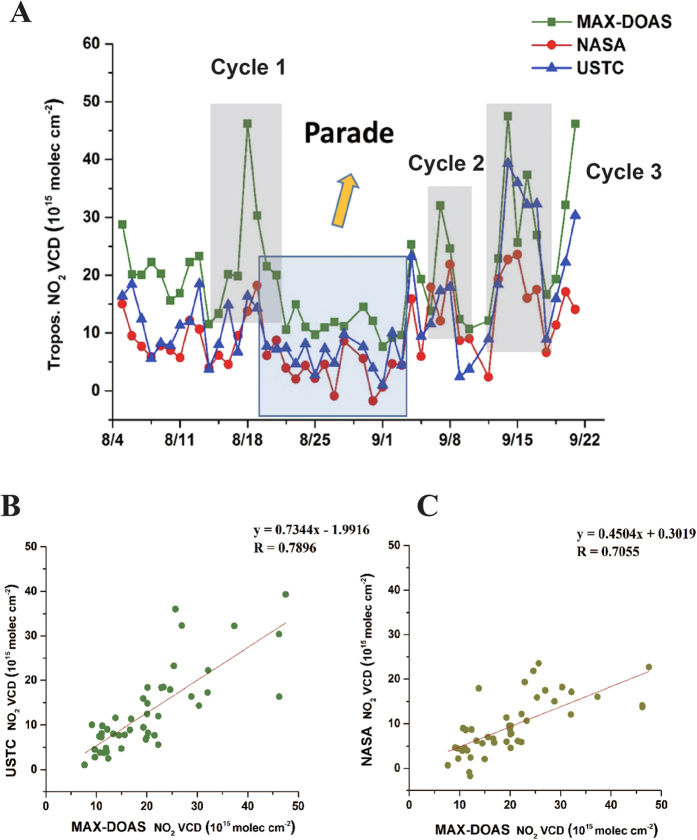
Time series (**A**) and correlation (**B,C**) of daily averaged tropospheric NO_2_ VCDs in Beijing urban area during the periods around Parade. MAX-DOAS data (green square curves) are temporally averaged around the NASA OMI and USTC OMI overpass time (red circle curves and blue delta curves, respectively). While the OMI data are spatially averaged over the grid cells within 30km of the ground location around the Beijing urban area. The transparent blue square area represents the Parade period. And the grey shade square areas separately represent “Cycle 1” (Aug. 14^th^ to 21^st^), “Cycle 2” (Sep. 6^th^ to 9^th^) and “Cycle 3” (Sep. 12^rd^ to 18^th^) of MAX-DOAS results.

**Figure 2:**
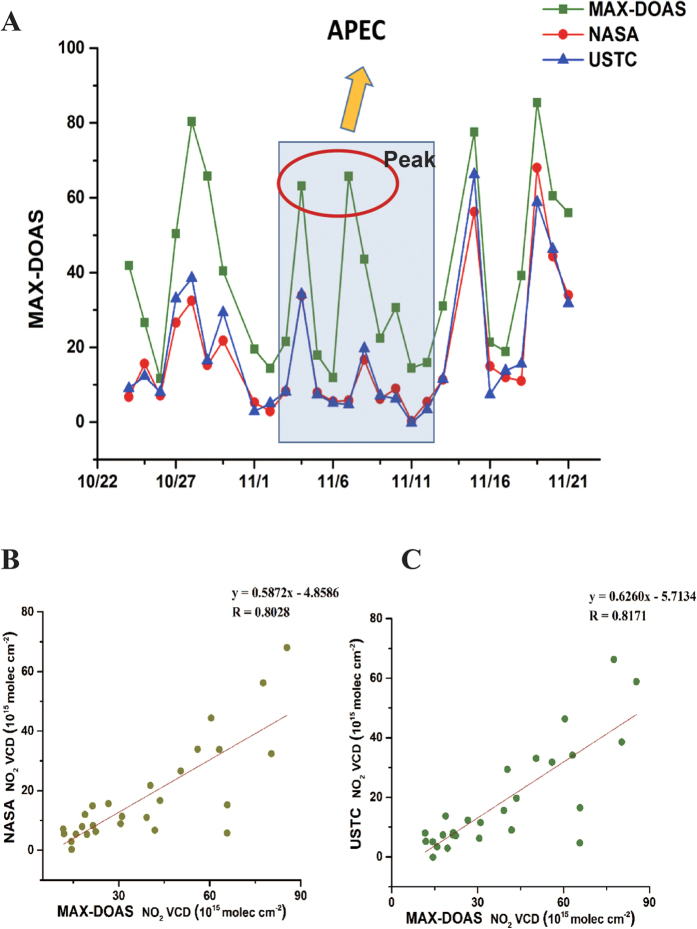
Time series (**A**) and correlation (**B,C**) of daily averaged tropospheric NO_2_ VCDs measured by the MAX-DOAS and OMI satellite in Beijing urban region during the periods around APEC. The transparent blue square area represents the APEC period. And the red circle represents the two peak values during the APEC period.

We use the retrieved NO_2_ VCD from MAX-DOAS measurements to analyze the temporal variation in urban Beijing (40°N, 116°22′48″E) for these two events (Fig. S1). In order to compare and validate with the OMI satellite data, the MAX-DOAS data are temporally averaged for the OMI satellite overpass time. Firstly, the NASA’s OMI tropospheric NO_2_ data products are employed, which are widely used in previous studies^5^,^19^. However, in view of the tropospheric air mass factors (AMF) of NASA’s OMI tropospheric NO_2_ products are calculated based on the monthly mean NO_2_ profile shapes derived from the Global Modeling Initiative (GMI) chemistry transport model multiannual simulation^19^,^23^, which might not be fully representative to the situation in China, especially in Beijing. So we also utilize the USTC’s NO_2_ products which account for the local atmospheric conditions and use the WRF-Chem model with measured climatology parameter^24^ and newest emission inventory^25^ to simulate trace gas profiles. The time series and inter-comparison of three independent tropospheric NO_2_ products are shown in Figs 1 and 2.

Generally, these three independent datasets showed a good agreement of during both Parade and APEC. A common trait, the NO_2_ VCDs had been abruptly decreased, was found during the Parade and APEC periods. It should be noted that the USTC OMI NO_2_ results present better correlation (*r* = 0.79 of Parade and *r* = 0.82 of APEC) than NASA OMI’s (*r* = 0.71 of Parade and *r* = 0.80 of APEC) with ground-based MAX-DOAS measurement, due to the corresponding local trace gases profiles used in AMF calculation, and the OMI NO_2_ SCD between USTC’s and NASA’s are quite close. Applying inappropriately trace gas profile to calculate AMF can cause up to 40% bias in previous study^26^.

However, from Table 1, we can find the MAX-DOAS measurements are systematically higher than the NASA and USTC OMI results. This systematic underestimation for OMI observations, which was also found in previous studies^18^,^19^, might be due to two main reasons. First, the grid cells’ values of satellite observations might be not only contained strong emission sources areas for the research site, but also average over neighboring cleaner areas^18^. Second, the different profile of NO_2_ and aerosols could cause a systematic underestimation of the real tropospheric NO_2_ VCDs in the satellite retrievals^18^,^19^. Previous studies show that the OMI NO_2_ columns were increased by 15–20%^27^,^28^, even up to 40% when a better estimated NO_2_ profile was applied in the AMF calculation for the NO_2_ column retrieval. Thus consistently in Figs 1 and 2, we found the difference of NO_2_column between USTC OMI results and MAXDOAS results are systematically 17% smaller than that of NASA’s, due to adopting newest emission inventory^25^ and measured local atmospheric conditions in AMF simulation.

As shown in Table 1, the averaged NO_2_ VCDs measured by MAX-DOAS instruments were respectively 22.07, 12.50 and 25.00 (10^15^ molec cm^−2^) during the periods around Parade. Compared with the pre-Parade, the averaged NO_2_ VCDs during Parade exhibits a distinct reduction ratio about 43% ((pre-Parade – Parade)/pre-Parade). While the air quality was observed to rapidly plummet from the perspective of NO_2_ with the end of Parade. The averaged NO_2_ VCDs during post-Parade was twice than compared to Parade ((post-Parade – Parade)/Parade). In the same way, during the periods around APEC, the mean NO_2_ VCDs measured by MAX-DOAS were respectively 39.01, 30.72 and 48.76 (10^15^ molec cm^−2^). 21% ((pre-APEC – APEC)/pre-APEC) and 59% ((post-APEC – AEPC)/APEC) were separately represented the decrease of APEC (compared with pre-APEC) and the growth of post-APEC (compared with APEC). The tremendous reductions during both two events (Parade and APEC) demonstrate the Chinese stringent control policy had been worked effectively from the perspective of NO_2_ for Beijing local.

**Figure 3:**
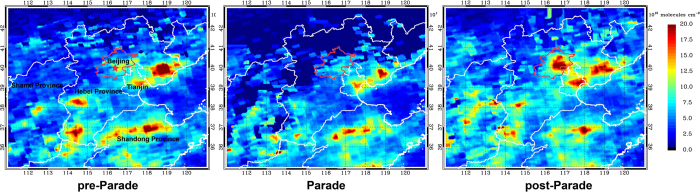
The spatial distribution of averaged tropospheric NO_2_ VCDs (unit: 10^15^ molec cm^−2^) during three periods around Parade. The USTC’s OMI tropospheric NO_2_ products were used here. The red outline represents Beijing city, and the red five-pointed star indicates the MAX-DOAS observation site at Beijing urban area (40°N, 116°22′48″E). This figure was generate by IDL 8.2 (http://www.esrichina.com.cn).

However, the NO_2_ results of MAX-DOAS could only reveal the variations around the site of Beijing urban areas during these two events. To more explicitly explore the distributions and variations in tropospheric NO_2_ we propose to use the USTC’s NO_2_ products retrieved from the OMI to analyze in this study, which showes a quite good correlation with MAX-DOAS results. Figure 3 presents the NO_2_ distributions in Beijing and its surrounding regions (including Hebei, Shanxi, Shandong Province and Tianjin Municipality) during three periods around Parade. Mean values were calculated in all of the three periods, i.e. pre-Parade, Parade and post-Parade, respectively. The spatial distributions of tropospheric NO_2_ VCDs were substantially similar but still different in diverse time frames. High NO_2_ VCD appeared on similar areas, including Beijing urban areas, Tianjin, southern Hebei, major areas of Shandong, and parts of Shanxi. And these areas which referred above are heavily industrialized and thus suffered by more intense anthropogenic emissions^5^. Compared with pre-Parade, we can easily find a sharp decrease over the urban Beijing, southern Hebei (including Tangshan city), Shandong and Shanxi during the Parade period. This phenomenon indicates that the strict emission control measures implemented in Beijing (nearly 2,000 industrial firms, including petrochemical and cement plants, suspended or cut production in Beijing local) and surrounding Provinces during Parade (based on Chinese media reports, http://en.people.cn/n/2015/0907/c98649-8946581.html), which were even more stringent than APEC periods, did significantly decrease the NO_2_ concentrations and improve the air quality of urban Beijing and its surrounding regions. Compared with Parade period, most of the areas, including Hebei, Shanxi, Shandong, especially for urban Beijing, had a rapid increase of NO_2_ VCDs during the post-Parade without strict control measures (Fig. 3).

In contrast, the NO_2_ VCDs had an apparent reduction only in Beijing during APEC period, and NO_2_ VCDs were not having a obvious reduction over the other surrounding regions of Beijing compared with pre- and post- APEC^5^. The results also corroborated that strict emission control measures during APEC periods may be not strict enough or not work well in Beijing surrounding regions compared with Parade periods.

## Discussion

### Role of regional source and meteorological impacts

Figure 1 show the time series of daily mean tropospheric NO_2_ VCDs about Parade. The result showed an intriguing phenomenon that the NO_2_ columns varied with time like a cycle. In the continual circulation, NO_2_ columns could be increased abruptly in someday and sustain for one day or several days, and dropped sharply for a few days. We named this phenomenon as “fluctuation effect”. The NO_2_ columns kept the significant fluctuation and displayed several peak values and valley values during pre-Parade and post-Parade period. While the significant fluctuation (peak and valley of NO_2_ columns) did not occur and the values are staying on relatively low levels during Parade period, which might be the effect of emission controlling measures.

In order to determine the formation of “fluctuation effect”, we pick out three typical cycles named “Cycle 1” (From August 14^th^ to 21^st^), “Cycle 2”(From September 6^th^ to 9^th^) and “Cycle 3” (From September 12^rd^ to 18^th^)respectively (Fig. 1). To find the trigger of peak and valley values, we analyzed the MAX-DOAS data during “Cycle 1” period by the cluster analysis of the 24-h air mass back trajectories (AMBTs) starting at 500 m at Beijing urban area (Fig. S2). Combined Fig. 1 with Fig. S2, we could find a close relationship between the directions of AMBTs and the “fluctuation effect”. The directions of AMBTs changed from north to south with the increasing of NO_2_ values (Fig. S2). Analogously, the NO_2_ values would be decreased when the directions changed from south to north. For instance, on August 14^th^ and 15^th^, the NO_2_ columns (11.55 and 13.37 10^15^ molec cm^−2^, respectively) were in the bottom position of “Cycle 1”, and the all AMBTs’ direction were from the north or north-west by chance. The NO_2_ columns (20.12 and 19.82 10^15^ molec cm^−2^, separately) on August 16^th^ and 17^th^ were increased compared with two days before, and the AMBTs’ paths were also transited from north to south. Just like 70.8% AMBTs were from southwestward on August 16^th^, and the 83.3% AMBTs’ routes were from south or southwest on August 17^th^. As we expected, 95.8% AMBTs were from south or southwest at the peak (46.22 10^15^ molec cm^−2^) on August 18^th^. As well, the process from the peak value down to the valley value experienced a period of transition (August 19^th^) which had a change from 4.2% west air mass increased to the 37.4% northwest air mass comparing with the “Peak” (August 18^th^). The corresponding NO_2_ columns (30.33 10^15^ molec cm^−2^) were also lower than peak value. The valley values (August 20^th^ and 21^st^) were appearing with the air mass directions all changed to the north. This result indicates the contamination of Beijing urban area was directly affected by the pollution transmission of Beijing south areas.

Similar results were also found in “Cycle 2” and “Cycle 3” (Figs S3 and S4). We could see the valley concentrations’ air mass came from the north or northeast, and the peak values’ air mass came from south or southwest. There’s a special point that the 62.5% air mass were surrounded by the south outsikts and peripheral cities of Beijing on September 7^th^. We can find the air mass were mainly from nearby southern Beijing during this day and their sojourn time of southern Beijing were more than the other days. It also meaned the Beijing area was not only influenced by the relatively distant southern cities, but also specially affected by southern neighboring areas of Beijing (just like the adjacency between Beijing and Hebei Province).

Figure 2 plots the time series of daily averaged tropospheric NO_2_ VCDs during the APEC periods. In contrast with Parade period, two peak values (Nov. 4^th^and 7^th^) were emerged during APEC period which was implementing the strict emission control measures period. We analyze the corresponding MAX-DOAS data by the cluster analysis (Fig. S5). There’re separately 79.2% and 83.3% AMBTs from south (Baoding, Langfang and Tianjin) on Nov. 4^th^and 7^th^. From the previous study^5^, we could find the regions of Tianjin and Hebei were still at a high level of NO_2_ during APEC period, which could have an influence on transporting to Beijing.

As suggested, meteorological conditions and regional atmospheric transport should play a key role in affecting the column NO_2_ levels. The NO_2_ peak values of “fluctuation effect” were mainly influenced by the polluted southern air mass, and the reason which led to the NO_2_ valley values is the clean effect of northern air mass. It also means the NO_2_ pollution of Beijing was directly affected by the southern surrounding areas’ atmospheric transport during the period without strict emission control measures.

### Identified the potential sources by PSCF

To further demonstrate the regional impact, we have analyzed the averaged NO_2_ VCDs measured by MAX-DOAS through the PSCF analysis in this study.The distributions of PSCF values in Beijing urban before, during and after the Parade are respectively shown in Fig. S6. For the pre-Parade and post-Parade period, due to the NO_2_ at high levels during these two periods the high PSCF values’ areas may represent the quintessentially potential emission sources for NO_2_ in Beijing urban. Both of these two periods show that cells with high PSCF values appeared mainly in the Beijing south suburbs and the southwestern surrounding cities around Beijing. The southern cells PSCF values were apparently higher than northern cells, which indicated the potential source areas maybe contain Baoding and Langfang and other southwestward cities around Beijing. That also proved the air pollutant transport from the locations nearby Beijing rather than farther places. For the Parade period, even though the NO_2_ VCDs were kept in a relatively low level during this period, we can also find there were some potential sources in Langfang and Tianjin according to Fig. S6. Synthesizes the above analysis, there is a common trait which indicates the NO_2_ pollution of Beijing urban during three periods around Parade were mainly affected by southern outskirts of Beijing, southwestern and southeastern surrounding cities of Beijing (e.g. Liulihe for Beijing and Baoding, Langfang for Hebei Province, as well as Tianjin).

Moreover, we referred to many series of air pollutants data (http://pm25.in/) for determining the air conditions about southern cities of Beijing, including AQI, PM_2.5_, PM_10_, NO_2_, O_3_, SO_2_ and so on. There are more than fifteen hundred sites on a nationwide scale in this website, and we selected 100 sites of data by sequence to present the pollutant concentrations during pre-Parade and post-Parade in this study (Tables S1 and S2). We found the air pollution (e.g. AQI, PM_2.5_, NO_2_ and O_3_) of Baoding and Langfang sites were at a quite high level compared to other sites. Especially for Baoding, the concentrations of AQI, PM_2.5_ and PM_10_ of 6 Baoding sites were always at the top 20 level for the total 1500s sites, some even at the top 10 level. The concentrations of NO_2_, O_3_ at the top 100 level for all sites. The averaged concentrations of AQI, PM_2.5_, PM_10_, NO_2_, O_3_, SO_2_ of the 6 sites for Baoding were separately 131.04, 93.92 ug m^−3^, 164.57 ug m^−3^, 35.18 ug m^−3^, 92.48 ug m^−3 ^and 17.27 ug m^−3^ much more than mean concentrations for total (60.27, 35.66 ug m^−3^, 66.70 ug m^−3^, 23.06 ug m^−3^, 66.11 ug m^−3^and 15.63 ug m^−3^, respectively). These data revealed the Baoding, Langfang and some other southern areas of Beijing were under high pollution levels.

From the above, we can confirm that the NO_2_ pollution in Beijing was mainly affected by the regional transport from the southern surrounding cities around Beijing (like Baoding, Langfang and Tianjin) which are the most potential NO_2_ sources areas for Beijing. The ambient contamination “fluctuation effect” for NO_2_ in Beijing was triggered by air mass directions. During the Parade, the controlling successfully decreased the impact of such regional source impact on NO_2_ in Beijing.

We also analyzed the averaged NO_2_ VCDs measured by MAX-DOAS through the PSCF analysis method during the periods around APEC (Fig. S7). It also revealed that high PSCF values with cells during pre-APEC were appeared on the southeastern suburbs of Beijing and southeastern surrounding cities of Beijing (just like Langfang and Tianjin). During the APEC period, the southeastern suburbs of Beijing and southeastern surrounding cities of Beijing (Langfang and Tianjin) were mainly source areas. Southern suburbs of Beijing, Baoding and Langfang were the primarily potential source areas during post-APEC. In account of the NO_2_ columns were slightly different between pre-APEC period and post-APEC period, we also combined the MAX-DOAS data of pre-APEC and post-APEC period and took a PSCF analysis (Fig. S7). Which could also reveal the consequence similar to above. In general, we could confirm the NO_2_ pollution in Beijing urban during the periods around APEC were mainly affected by the emission from southern and southeastern suburbs of Beijing, southern and southeastern cities of Beijing, especially from Tianjin, Langfang and Baoding.

### Controlling impact on NO_2_

**Figure 4:**
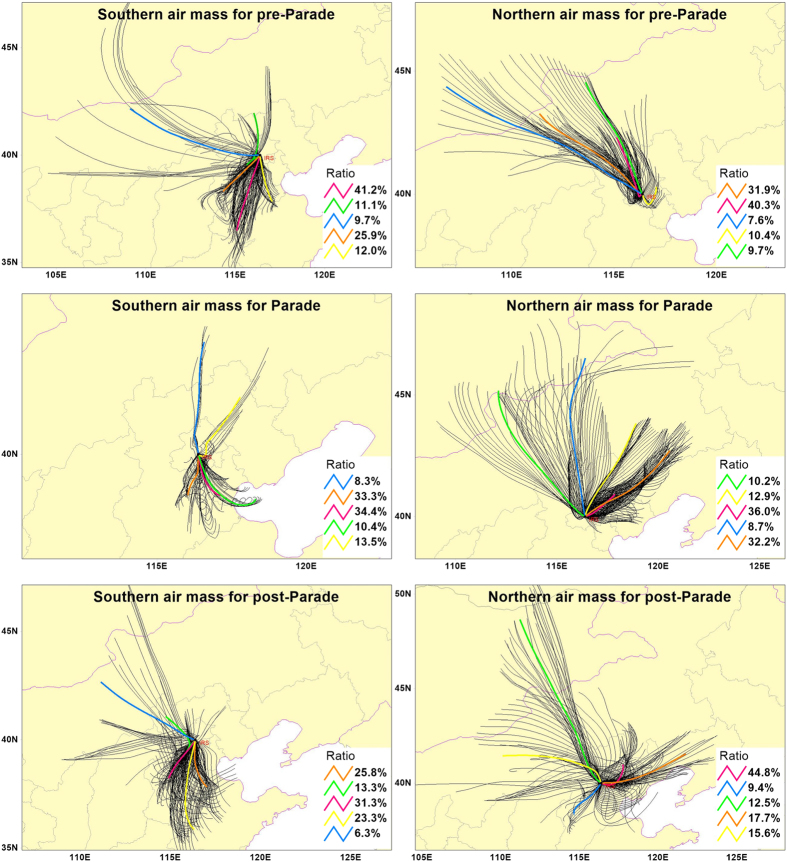
Cluster analysis of the 24-h AMBTs for southern and northern air mass during pre-Parade, Parade and post-Parade starting at 500m in Beijing urban area. The southern air mass means that the AMBTs were almost from south, southeast and southwest (>50%) for a whole day. Likewise, northern air mass means more than 50% AMBTs were from north, northeast and northwest. The black lines mean the AMBTs during the certain period for every hour (which have been signed in title). And the color lines present the 5 categories directions of AMBTs. Base map is from TrajStat 1.2.2 software (http://www.meteothinker.com).

**Figure 5:**
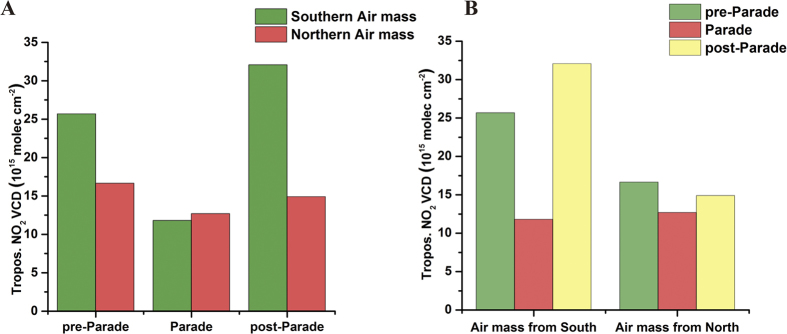
The comparison of mean tropospheric NO_2_ VCDs by different air mass during the same period (**A**). Similarly, the comparison of mean tropospheric NO_2_ VCDs by same air mass during the diverse periods (**B**).

According to the foregoing analysis, the NO_2_ columns would be seriously influenced by air mass in Beijing. Previous studies showed meteorological conditions were found to play a significant role in reducing pollution levels during the Olympic Games^29^,^30^,^31^,^32^. These results reflect the uncertainties in assessing the impact of emission controls on air pollutants over a certain area. For avoiding the influence of the meteorological factor, we had performed the cluster analysis of the 24-h air mass back trajectories for the periods around Parade which from August 5^th^ to September 21^st^. And for better contrast, we divide these AMBTs into two types during three periods around Parade (Fig. 4, Table S3): 1. Southern air mass, it means that the AMBTs were almost from south, southeast and southwest (>50%) for a whole day. 2. Northern air mass, which means more than 50% AMBTs were from north, northeast and northwest for a full day. From the Table S3 and Fig. 5, we compared the southern air mass with northern air mass, and could easily find the mean tropospheric NO_2_ VCDs for southern air mass during both pre-Parade and post-Parade period (25.68 and 32.07 10^15^ molec cm^−2^) were 2~3 times higher than northern air mass during these two periods (16.65 and 14.90 10^15^ molec cm^−2^). These huge differences directly showed the importance of meteorological factors and southern regional atmospheric transport. Baoding, Langfang and other southern cities from Beijing were suffered more serious air pollution than Beijing (Table S1), and much more air pollution would be brought in Beijing local when the air mass passed through these areas. Compared with southern regions around Beijing, the northern areas were kept in a cleaner conditions. However, with a series of emission control policy implemented in Beijing and its southern surrounding cities during Parade period, the impact of air mass had been vanished. The mean tropospheric NO_2_ VCDs for southern air mass during Parade (11.80 10^15^ molec cm^−2^) was even a little lower than northern air mass (12.69 10^15^ molec cm^−2^), and both of them stayed at a relatively low level. This phenomenon reflected the effectiveness of strict emission control measures. For southern air mass, compared with averaged NO_2_ VCDs during pre-Parade (25.68 10^15^ molec cm^−2^), the averaged NO_2_ VCDs during Parade (11.80 10^15^ molec cm^−2^) had been decreased about 54%. The averaged NO_2_ VCDs during post-Parade (32.07 10^15^ molec cm^−2^) was almost 3 times higher than during Parade period. These results directly proved the southern peripheral cities around Beijing plays a key role in pollutants transport for Beijing. This abruptly diminishing during Parade manifests the real effect of emission control measures and also shows the importance of emission controls which were implemented in cities to the south of Beijing. For northern air mass, the averaged NO_2_ VCDs were respectively 16.65 and 14.90 10^15^ molec cm^−2^ during pre-Parade and post-Parade period, both of which are a little more than during Parade (12.69 10^15^ molec cm^−2^). And all of them stayed at a relatively low level for NO_2_ pollutants, that means the regions surrounding north of Beijing were much cleaner than south. We haven’t found a significant effect of transmission for northern air mass, and there were fewer emission controls implemented for the north and northwest of Beijing compared with southern regions. The NO_2_ VCDs for the northern air mass had a little decrease during Parade period compared with pre-Parade period, which represents the probable effect of Beijing owns’ emission measures due to eliminating the influence of the southern transmissions. However, according to the results of above, limiting the cities to the south of Beijing may be more important than Beijing local.

### Change in O_3_ versus NO_2_ during and around the period of APEC and Parade

In order to explore the chemical sensitivity of PO_3_ during Parade periods and APEC periods in Beijing, we used the USTC OMI tropospheric NO_2_ products and USTC recalibrated OMI total HCHO products to calculate the Ratio over Beijing and surrounding areas during these two events. And we also combined the Ratio and the corresponding variations of NO_2_ and HCHO to analyze how O_3_ varied as the function of Ratio.

**Figure 6:**
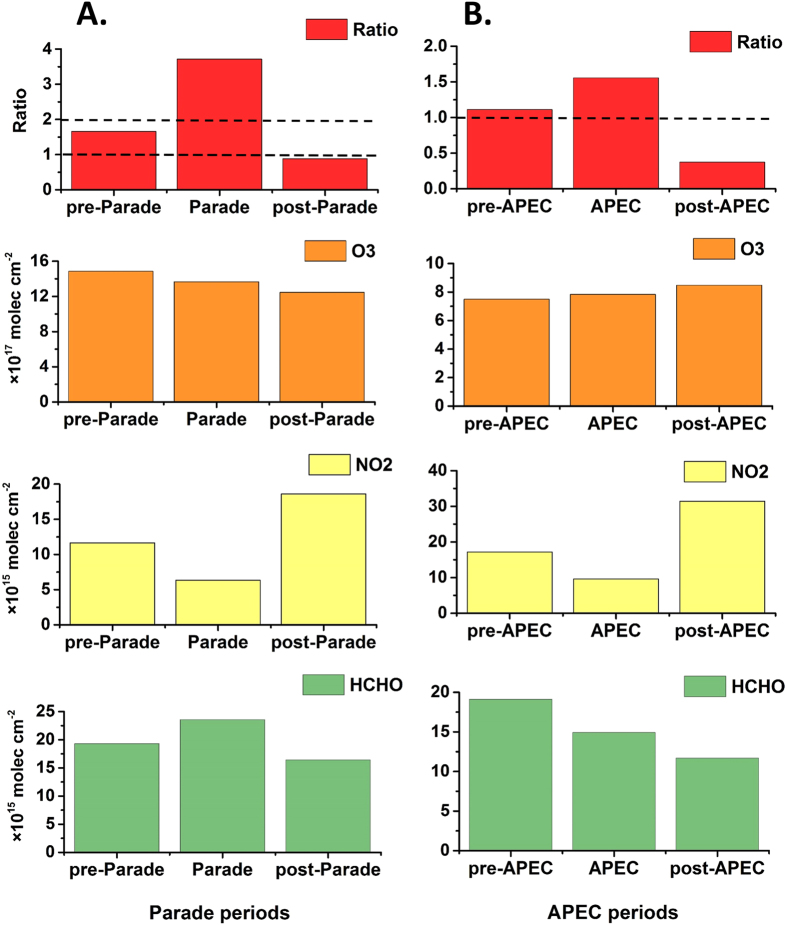
The variations of averaged Ratio and mean tropospheric NO_2_, O_3_ and HCHO VCDs of three periods during Parade (**A**) and during APEC (**B**) at Beijing urban site (40°N, 116°22′48″E)within 1° × 1° mean value. The USTC’s OMI tropospheric NO_2_ products, USTC’s recalibrated OMI HCHO products and OMI tropospheric O_3_ products were used here.

From Fig. 6A, we can easily find the Ratio is between 1 and 2 (1.76) at Beijing urban site within 1° × 1° mean value (40°N, 116°22′48″E) during pre-Parade, which indicates this area was at a mixed VOC-NO_x_-limited regime. With a series of strict emission control measures during Parade, the Ratio had changed from 1.65 to 3.71. It means the PO_3_ conditions had also changed from mixed VOC-NO_x_-limited to a predominantly NO_x_-limited condition due to the sharp drop of NO_2_ during Parade. It is observed that the tropospheric averaged O_3_ VCDs had a decline (from 14.84 10^17^molec cm^−2^ during pre-Parade to 13.65 × 10^17^molec cm^−2^ during Parade) from the pre-Parade period transited to the Parade period, which was maybe caused by the rapid decrease of local NO_2_ because the PO_3_ was exactly stayed at NO_x_-limited chemistry.

After the strict emission control measures were lifted, the NO_2_ returned to relatively high values as pre-Parade and the Ratio was also diminished (Ratio = 0.90, <1), which indicated the PO_3_ was turned into a VOC-limited condition during post-Parade. At this point, the reduction of mean HCHO VCDs, which was the normal seasonal decrease of HCHO, maybe was the primary reason caused by the O_3_ kept decreasing correspondingly.

**Figure 7:**
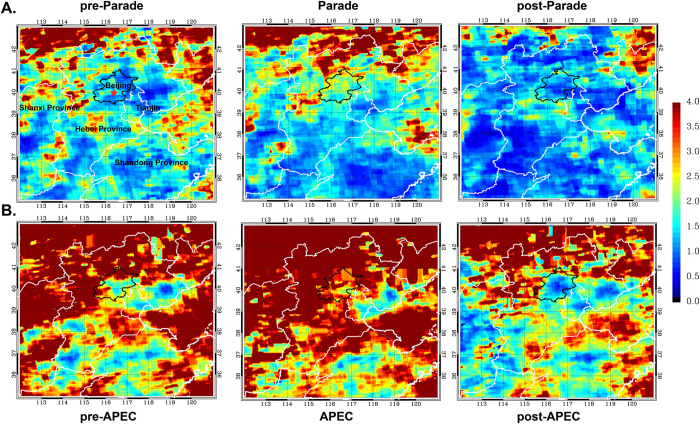
The averaged Ratios over Beijing and its surrounding regions during three periods of Parade and APEC. The black outline represents Beijing city, and the red five-pointed star indicates the MAX-DOAS observation site at Beijing urban area (40°N, 116°22′48″E). This figure was generate by IDL 8.2 (http://www.esrichina.com.cn).

Figure 7A shows the spatial variation of the Ratios over Beijing and surrounding areas during three periods around Parade. During pre-Parade, we found that most of Beijing urban areas were stayed at mixed VOC-NO_x_-limited (1< Ratio <2) and part of eastern, southwestern and southern Beijing were stayed at both VOC-limited and mixed VOC-NO_x_-limited. And the other regions were presented to the NO_x_-limited. When the time was entering the Parade period, PO_3_ was shifted to a predominantly NO_x_-limited regime (Ratio >2) including Beijing urban and a major part of southern and southwestern areas. These results reveal that Chinese control policies had been worked effectively from the perspective of NO_2_, however, HCHO had not an apparent decrease over these areas. And the ratio had been turned into VOC-limited regime over Beijing and surrounding regions because NO_2_ returned to relatively high values. The result which indicated PO_3_ is VOC-limited in urban Beijing is also consistent with previous studies^33^,^34^.

Above of all, the emission control measures during Parade were not only work effectively for regional NO_2_ pollution control patterns but also effective for O_3_ controlling.

Similarly, Fig. 6B shows the Ratio and variations of O_3_, NO_2_ and HCHO averaged VCDs at Beijing urban site within 1° × 1° mean value (40°N, 116°22′48″E) during three periods around APEC. It is obviously that both pre-APEC and APEC were stayed at a mixed VOC-NOx-limited condition. From the pre-APEC to APEC, both NO_2_ and HCHO had a certain reduction which should lead the O_3_ diminishing. Conversely, the mean O_3_ VCDs were increased slightly instead of decline. Interestingly, similar results were also occurred to the previous measurements which were probably caused by the decreasing NO-titration of ozone (i.e. NO+O_3_→NO_2_+O_2_) and regional transport of photochemically aged air^20^,^35^. And the similar results (the mean O_3_ VCDs were increased slightly) were also presented to post-APEC, while the HCHO was decreased during VOC-limited condition. This result indicates that emission controls in this case maybe not strict enough or worked well to lessen the levels of ozone^20^.

We can find the spatial variation of the Ratios over Beijing and surrounding areas during three periods around APEC from Fig. 7B. Compared with pre-APEC, the Ratio was changed from around 0~1.5 (VOC-limited and mixed VOC-NO_x_-limited) to around 1~2.5 (mixed VOC-NO_x_-limited and NO_x_-limited) in Beijing areas during APEC due to the rapid NO_2_ decrease in this period. However, in other regions especially for Beijing southern areas the Ratio was presented to a decreasing tendency, which indirectly reflects the NO_2_ VCDs were even increased in the areas neighboring Beijing. It also suggests that the emission control measures were not effective to Beijing surrounding regions during APEC period. After the emission control measures lifted, the Ratio had a dramatically decrease overall due to the diminishing of HCHO and NO_2_ growing broadly.

In summary, even though both Parade and APEC period had a series of strict emission control policies, the control effect of Parade was far more than APEC. The emission control measures of Parade were not only limited the NO_2_ pollutant including Beijing and from outside adjacent areas but also suppress the O_3_ contaminant. However, during the APEC the emission control measures had not been worked so effective for surrounding areas. These results and the additional information from Ratio may help government formulate some appropriate pollution control strategies.

## Method

### MAX-DOAS

The MAX-DOAS experimental instrument is undertaken in the site (40°N, 116°22′48″E) which is located in the northwest of Beijing urban area, nearby the fourth Ring Roads from October 2014 to September 2015.

MAX-DOAS is a passive DOAS approach based on measurements of scattered sunlight at different elevation angles and zenith towards the horizon, and it could retrieve the NO_2_ column densities by the DOAS algorithm^11^. The MAX-DOAS instrument at this site was collected by sequential measurements which were made at 8 different elevation angles (3°, 5°, 8°, 10°, 12°, 20°, 30° and 90°) of scattered sunlight. The Fraunhofer reference (FRS) was used to remove the solar Fraunhofer structure in the scattered sunlight, which was usually selected at the 90° elevation angle during noon of a clear day^11^. The QDOAS software^36^ (http://uv-vis.aeronomie.be/software/QDOAS/) with NO_2_ retrieval settings of MAX Plank institute for Chemistry (MPIC) (http://joseba.mpch-mainz.mpg.de/mad_analysis.htm) was applied to analyze the spectra between 338 and370 nm. Before the spectral analysis, the effects of electronic offset and dark current are also removed by spectra measured at the same condition.

Finally, differential slant column density (DSCD) were obtained from the QDOAS software outputs, which means the slant column density (SCD) between the measured spectrum and the FRS. The tropospheric DSCDα can be expressed as (α: denotes the elevation angle):


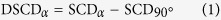


The SCD is also influenced by the length of light path and the observation geometry, thus it needs to be converted to the vertical column density (VCD) which is not affect by the observation geometry and light path and can be used to compare different measurements. The VCD is calculated with the air mass factor (AMF):


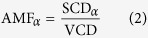


According to the above equation, the SCDs at the angles of 90° and α can be described as:


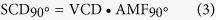



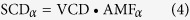


By substituting equalities (3) and (4) into equality (1), we can acquire^18^:


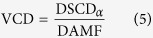


Where the DAMF is the differential atmospheric air mass factor:


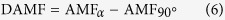


In this study, thetroposphericAMFs were calculated using the Vector Linearized Discrete Ordinate Radiative Transfer (VLIDORT) model. To account for local atmospheric conditions, WRF-Chem model with measured climatology parameter^24^ and newest emission inventory^25^ is used to generate the trace gas profiles for MAX-DOAS AMF calculation.

### The Ozone Monitoring Instrument (OMI)

The OMI is an urltraviolet/visible (UV/VIS) passive nadir-viewing imaging spectrometer, which is placed on the Earth Observing System’s (EOS) Aura satellite. The EOS Aura satellite was launched on 15 July 2004 and approximately 14.5 sun synchronous polar orbits are scanned each day, which could provide daily global maps. The spatial resolution of OMI is 13 km × 24 km (around 320 km^2^) at nadir and increases to about 40 km × 160 km (6400 km^2^) at both edges of the track in the global observation mode. A scan (60 synchronously measurements) corresponds to around 2600 km across one orbital track direction.

In this study, both NASA’s and USTC’s OMI tropospheric trace gases products are used. For NO_2_, the NASA’s OMI tropospheric NO_2_ products (http://disc.sci.gsfc.nasa.gov/Aura/), which use monthly mean NO_2_ profile from Global Modeling Initiative (GMI) chemistry transport model^23^ for VCD column conversion. USTC tropospheric NO_2_ SCDs, which are retrieved by the DOAS^11^ trace gas fitting algorithm using a nonlinear least-squares (NLLS) inversion technique from the OMI spectra. Consistent with MAX-DOAS measurements in study, to account for local atmospheric conditions, WRF-Chem model with measured climatology parameter and newest emission inventory^25^ is also used to simulate trace gas profiles for VCDs products conversion from former calculated SCDs. For OMI HCHOVCDs, we recalculated them from the SCDs of NASA HCHO products^37^ based on WRF-Chem model simulated profiles to account for local atmospheric conditions. The OMI tropospheric O_3_ products^38^ are download from (http://disc.sci.gsfc.nasa.gov/Aura/).

### WRF-Chem model and Emission Inventory

The numerical model adopted in this study is WRF-Chem version 3.7, which is an online-coupled chemical transport model considering multiple physical and chemical processes, including emissions and deposition of pollutants, advection and diffusion, gaseous and aqueous chemical transformation, aerosol chemistry and dynamics^39^. It is capable of simulating atmospheric chemistry on a regional scale and has been successfully applied in several of our previous studies^40^,^41^. In this work, the model domain covered East China and its surrounding area, centering at 35.0°N, 110.0°E with a 20 × 20 km grid resolution, as demonstrated in Fig. S8. There are 27 vertical layers from the ground level to the top pressure of 50 hPa. The 6 hourly Final operational global analysis (FNL) data with a 1° × 1° spatial resolution produced by the National Centers for Environmental Prediction (NCEP) was used as initial and boundary conditions of meteorological fields. In addition, NCEP’s ADP global upper-air observations (NCAR archive ds351.0) were assimilated every 6 hours to enhance the meteorology reproduction. Key physical parameterization options for the modelling were the Noah land surface scheme to describe the land-atmosphere interactions^42^, the Lin microphysics scheme^43^ with the Grell cumulus parameterization to reproduce the cloud and precipitation processes^24^, the YSU boundary layer scheme, and the RRTMG short- and long-wave radiation scheme^44^.

For the numerical representation of atmospheric chemistry, we used the CBMZ (Carbon-Bond Mechanism version Z) photochemical mechanism combined with MOSAIC (Model for Simulating Aerosol Interactions and Chemistry) aerosol model^45^,^46^. Both natural and anthropogenic emissions were included for the regional WRF-Chem modelling in the present work. Typical anthropogenic emissions were obtained from the Multi-resolution Emission Inventory for China (MEIC) database^25^, in which emissions sources were classified into five main sectors: power plants, residential combustion, industrial processes, on-road mobile sources, and agricultural activities. This database covered most of anthropogenic pollutants, such as SO_2_, NO_x_, CO, volatile organic compounds (VOCs), NH_3_, PM, BC, and OC. The biogenic VOC and NO emissions were calculated online by using the Model of Emissions of Gases and Aerosols from Nature (MEGAN) embedded in WRF-Chem^47^. More than 20 biogenic species, including isoprene, monoterpenes (e.g., α-pinene and β-pinene) and sesquiterpenes, were considered and then involved in the photochemistry calculation.

The simulation was conducted for 20 September to 20 November 2014 (Fig. S9), and 1 August to 26 September 2015 (Fig. S10), during which each run covered 24 hours. The chemical outputs from the preceding run were used as the initial conditions for the next run. First two weeks were regarded as the model spin-up period, so as to minimize the influences of initial conditions and allow the model to reach a state of statistical equilibrium under the applied forcing^48^.

### Cluster analysis and PSCF analysis

In this study, the 24h air-mass back trajectories arriving at Beijing urban site (40°N, 116°22′48″E) 500 m above ground level (AGL) were calculated every hour for each day, which were computed by the Hybrid Single-Particle Lagrangian Integrated Trajectory (HYSPLIT) model (http://ready.arl.noaa.gov/HYSPLIT.php) of National Oceanic and Atmospheric Administration (NOAA)^49^,^50^. The AMBTS could be used to identify the transport pathways of pollutants and potential source regions by the calculating Lagrangian path of air parcels in the chosen time scale^51^.

Cluster analysis is based on the AMBTs through statistical analysis, which could be proposed as a useful way to assess the potential sources of ambient^52^. In this study, cluster analysis was served for the periods both Parade and APEC through the software TrajStat^53^ (http://www.meteothinker.com). The potential source contribution function (PSCF) analysis has been frequently used to identify the suspicious locations of emission sources that influence pollutant concentrations at the receptor site^54^,^55^,^56^,^57^,^58^, which is based on the estimates of the motion of AMBTs in time with contamination density measured at the receptor site. In this study, the AMBTs were distributed with the cells of 0.2° × 0.2°resolution grid. And the grid cells PSCF values were computed by counting the trajectory section endpoints terminating within each cell, including trajectories which are not only ending at the cell but also passing through the cell. The PSCF value could be described as:


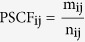


Where n_ij_ represents the total number of trajectory section endpoints that fills into the ij^th^ cell, and m_ij_ is the number of section endpoints in the identical cell corresponding with trajectories associated with constituent values at certain receptor site surpassing a pre-specified criterion value^59^. In this study, the criteria values were the corresponding mean NO_2_ VCDs of MAX-DOAS at the receptor site. Hence, cells with high PSCF values suggest these areas are likely to produce high pollutant values at the receptor sites, so they are sufficiently deemed to be probable source regions. To diminish the uncertainty of PSCF resulted from small n_ij_ values, every PSCF value should be multiplied by an arbitrary weight function W_ij_^60^ to better represent the uncertainty in the values for which n_ij_ with small values. The weight function W_ij_ is defined as:


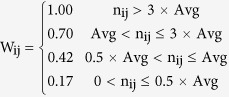


Where Avg presents the mean number of endpoints in each cell. The PSCF value would be reduced by the weight function when the sum of endpoints in a cell was less than around three times the mean value of the endpoints per cell^56^. In this study, the contributions of other atmospheric pollution source regions at Beijing urban site was identified by the PSCF analysis with the software TrajStat^53^.

## Additional Information

**How to cite this article**: Liu, H. *et al*. A paradox for air pollution controlling in China revealed by “APEC Blue” and “Parade Blue”. *Sci. Rep.* **6**, 34408; doi: 10.1038/srep34408 (2016).

## Supplementary Material

Supplementary Information

## Figures and Tables

**Table 1:** Tropospheric NO_2_ mean VCDs (in units of 10^15^ molecules cm^−2^) at Beijing urban area in different time periods.

Time period	MAX-DOAS	NASA-OMI	USTC-OMI
pre-Parade	22.07	9.18	11.65
Parade	12.50	3.81	6.21
post-Parade	25.00	14.24	19.44
pre-APEC	39.01	14.87	17.20
APEC	30.72	9.88	10.66
post-APEC	48.76	31.48	31.43
