# Supplementary material for: A paradox for air pollution controlling in China revealed by “APEC Blue” and “Parade Blue”
Source: Sci Rep. 2016 Sep 29;6:34408. doi: 10.1038/srep34408 (PMC5041090; doi:10.1038/srep34408)
Supplement: Supplementary Information [file srep34408-s1.docx]

Supplementary Materials for the manuscript:

**A paradox for air pollution controlling in China revealed by “APEC Blue” and “Parade Blue”**

Haoran Liu^1,3^, Cheng Liu^1,2,3*^, Zhouqing Xie^1,2,3*^, Ying Li^4^, Xin Huang^5,6^, Shanshan Wang^7^, Jin Xu^3^, Pinhua Xie^2,3^

1. School of Earth and Space Sciences, University of Science and Technology of China, Hefei, 230026, China

2. Center for Excellence in Urban Atmospheric Environment, Institute of Urban Environment, Chinese Academy of Sciences, Xiamen 361021, China

3. Key Lab of Environmental Optics & Technology, Anhui Institute of Optics and Fine Mechanics, Chinese Academy of Sciences, Hefei 230031, China

4. Division of Environment, Hong Kong University of Science and Technology, Kowloon, Hong Kong

5. Institute for Climate and Global Change Research & School of Atmospheric Sciences, Nanjing University, Nanjing, 210023, China

6. Collaborative Innovation Center of Climate Change, Jiangsu Province, China

7. Department of Atmospheric Chemistry and Climate, Institute of Physical Chemistry Rocasolano, CSIC, Madrid, 28006, Spain

Correspondence author:

[chliu81@ustc.edu.cn](mailto:chliu81@ustc.edu.cn) (C.L.); [zqxie@ustc.edu.cn](mailto:zqxie@ustc.edu.cn) (Z.Q.X.)

To complement the information given in the main manuscript, the following sections provide supporting information Tables S1, S2 and S3, and Figures S1, S2, S3, S4, S5, S6, S7, S8, S9 and S10.

**Supplementary Tables**

**Table S1.** **The pollutants daily mean concentrations of 100 different sites which were selected by sequence from the total 1500 sites over the country during pre-Parade. Unit of these pollutants is “ug m^-3^” except for AQI. The 1014A-1027A, 1051A-1056A and 1067A-1070A represent Tianjin sites, Baoding sites and Langfang sites, respectively.**

**Table S2.** **The pollutants daily mean concentrations of 100 different sites which were selected by sequence from the total 1500 sites over the country during post-Parade. Unit of these pollutants is “ug m^-3^” except for AQI. The 1014A-1027A, 1051A-1056A and 1067A-1070A represent Tianjin sites, Baoding sites and Langfang sites, respectively.**

**Table S3. Tropospheric NO_2_ mean VCDs (in units of 10^15^ molecule cm^−2^) measured by MAX-DOAS for air mass from South and North during the three periods at Beijing urban area in different time periods. Mean represent the averaged tropospheric NO_2_ for every period.**

**Table S1.**

| **Site** | **AQI(N/A)** | **PM_2.5_** | **PM_10_** | **NO_2_** | **O_3_** | **SO_2_** |
| --- | --- | --- | --- | --- | --- | --- |
| 1001A | 78.50 | 55.10 | 74.45 | 43.84 | 63.54 | 5.23 |
| 1002A | 63.86 | 41.47 | 52.98 | 16.94 | 86.77 | 4.24 |
| 1003A | 81.00 | 57.44 | 88.58 | 49.79 | 62.36 | 7.23 |
| 1004A | 74.87 | 52.00 | 67.49 | 49.14 | 56.60 | 7.34 |
| 1005A | 77.91 | 54.87 | 65.01 | 53.53 | 67.11 | 6.63 |
| 1006A | 79.61 | 56.02 | 84.11 | 47.77 | 67.78 | 6.06 |
| 1007A | 77.07 | 53.27 | 75.30 | 44.60 | 56.00 | 5.37 |
| 1008A | 75.92 | 53.76 | 62.69 | 39.63 | 50.87 | 3.47 |
| 1009A | 69.87 | 48.88 | 56.11 | 14.70 | 72.53 | 4.50 |
| 1010A | 67.95 | 46.45 | 49.35 | 38.73 | 69.58 | 4.79 |
| 1011A | 80.19 | 54.70 | 85.53 | 62.49 | 72.54 | 6.29 |
| 1012A | 79.65 | 51.29 | 88.68 | 37.85 | 68.35 | 4.67 |
| 1013A | - | - | - | - | - | - |
| **1014A** | **63.27** | **38.73** | **76.45** | **24.53** | **36.80** | **10.84** |
| **1015A** | **66.38** | **42.09** | **80.28** | **27.97** | **44.31** | **12.61** |
| **1016A** | **64.01** | **39.97** | **73.33** | **27.27** | **52.60** | **13.07** |
| **1017A** | **64.17** | **41.29** | **73.37** | **30.15** | **59.48** | **14.94** |
| **1018A** | **72.29** | **48.07** | **84.67** | **28.02** | **39.66** | **9.82** |
| **1019A** | **70.68** | **46.00** | **83.99** | **25.83** | **49.19** | **12.27** |
| **1020A** | **-** | **-** | **-** | **-** | **-** | **-** |
| **1021A** | **67.80** | **36.48** | **85.33** | **28.09** | **45.19** | **18.32** |
| **1023A** | **68.17** | **45.04** | **81.32** | **30.62** | **48.04** | **10.73** |
| **1024A** | **66.75** | **43.03** | **75.81** | **23.74** | **56.12** | **14.86** |
| **1025A** | **61.84** | **37.93** | **71.63** | **27.64** | **39.58** | **11.79** |
| **1026A** | **68.37** | **45.45** | **72.67** | **16.11** | **53.80** | **15.56** |
| **1027A** | **82.05** | **59.07** | **83.72** | **22.26** | **57.65** | **14.75** |
| 1028A | - | - | - | - | - | - |
| 1029A | 88.48 | 57.30 | 112.20 | 60.07 | 60.63 | 27.60 |
| 1030A | 76.59 | 43.42 | 93.53 | 44.16 | 58.75 | 35.70 |
| 1031A | 73.87 | 44.30 | 93.87 | 45.89 | 58.77 | 21.56 |
| 1032A | 88.19 | 44.67 | 112.28 | 43.44 | 51.82 | 22.44 |
| 1033A | 77.50 | 50.02 | 92.29 | 46.62 | 59.83 | 21.09 |
| 1034A | 58.86 | 34.80 | 67.11 | 41.32 | 51.55 | 17.66 |
| 1035A | 64.64 | 41.07 | 85.89 | 37.23 | 94.50 | 55.62 |
| 1036A | 66.17 | 33.58 | 79.56 | 59.68 | 66.94 | 37.93 |
| 1037A | 66.69 | 31.56 | 80.55 | 53.01 | 66.21 | 28.57 |
| 1038A | 63.91 | 33.25 | 79.23 | 46.85 | 57.22 | 46.17 |
| 1039A | 71.96 | 37.74 | 93.55 | 65.47 | 60.47 | 35.28 |
| 1040A | 67.28 | 37.33 | 80.61 | 52.08 | 64.71 | 39.67 |
| 1041A | 70.89 | 40.11 | 82.18 | 61.34 | 63.40 | 39.38 |
| 1042A | 31.63 | 14.59 | 30.82 | 26.86 | 38.04 | 28.19 |
| 1043A | 64.39 | 24.38 | 90.97 | 53.32 | 39.46 | 28.15 |
| 1044A | 55.33 | 20.82 | 68.10 | 34.65 | 39.47 | 15.14 |
| 1045A | 50.57 | 12.08 | 60.26 | 40.68 | 29.60 | 38.72 |
| 1046A | 46.83 | 16.40 | 53.67 | 34.88 | 15.91 | 23.25 |
| 1047A | 83.88 | 48.30 | 117.53 | 44.91 | 63.92 | 14.18 |
| 1048A | 77.98 | 48.52 | 94.21 | 24.05 | 59.16 | 20.41 |
| 1049A | 108.75 | 73.39 | 145.50 | 55.87 | 61.14 | 33.56 |
| 1050A | 85.60 | 59.26 | 98.27 | 37.85 | 48.79 | 30.13 |
| **1051A** | **95.95** | **66.64** | **118.50** | **50.75** | **78.99** | **15.50** |
| **1052A** | **100.62** | **74.09** | **120.08** | **41.79** | **68.98** | **19.58** |
| **1053A** | **87.72** | **60.06** | **100.15** | **43.20** | **66.65** | **13.21** |
| **1054A** | **91.06** | **63.27** | **114.12** | **42.36** | **79.87** | **15.67** |
| **1055A** | **96.80** | **62.76** | **130.02** | **44.89** | **44.88** | **10.73** |
| **1056A** | **105.65** | **77.02** | **134.13** | **44.67** | **71.40** | **16.65** |
| 1057A | 52.90 | 31.46 | 65.72 | 20.58 | 77.93 | 12.66 |
| 1058A | 57.06 | 36.16 | 63.55 | 21.59 | 88.73 | 9.78 |
| 1059A | 55.14 | 26.18 | 66.94 | 20.88 | 78.12 | 19.22 |
| 1060A | 62.26 | 40.85 | 80.00 | 25.73 | 58.81 | 12.97 |
| 1061A | 51.15 | 15.58 | 65.19 | 39.47 | 68.38 | 7.77 |
| 1062A | 57.58 | 30.08 | 67.60 | 43.15 | 51.28 | 11.45 |
| 1063A | 53.03 | 28.80 | 57.81 | 25.43 | 57.90 | 4.79 |
| 1064A | 56.91 | 32.27 | 62.60 | 32.19 | 63.85 | 10.79 |
| 1065A | 61.27 | 31.51 | 76.44 | 25.25 | 63.39 | 13.93 |
| 1066A | 55.02 | 34.27 | 50.81 | 13.14 | 57.58 | 5.80 |
| **1067A** | **87.14** | **55.43** | **113.00** | **63.08** | **65.73** | **11.71** |
| **1068A** | **-** | **-** | **-** | **-** | **-** | **-** |
| **1069A** | **85.73** | **53.86** | **115.35** | **49.32** | **76.12** | **13.32** |
| **1070A** | **79.67** | **53.93** | **86.12** | **48.41** | **69.36** | **11.87** |
| 1071A | 58.96 | 36.84 | 67.37 | 51.35 | 62.94 | 39.14 |
| 1072A | 58.05 | 35.96 | 63.44 | 38.78 | 75.11 | 32.68 |
| 1073A | 53.52 | 31.82 | 59.49 | 38.50 | 67.98 | 27.92 |
| 1074A | 104.36 | 70.56 | 136.07 | 44.56 | 82.65 | 26.22 |
| 1075A | 102.58 | 65.95 | 146.08 | 44.53 | 85.96 | 21.74 |
| 1076A | 106.18 | 76.84 | 120.10 | 41.00 | 87.87 | 22.56 |
| 1077A | 85.09 | 57.69 | 107.11 | 49.32 | 62.09 | 17.61 |
| 1078A | 85.27 | 52.60 | 117.78 | 55.62 | 38.85 | 35.18 |
| 1079A | 85.79 | 57.34 | 110.80 | 53.26 | 35.16 | 31.35 |
| 1080A | 71.85 | 46.61 | 85.31 | 54.24 | 55.31 | 28.81 |
| 1081A | 78.85 | 51.29 | 98.15 | 28.63 | 48.30 | 14.86 |
| 1082A | 89.40 | 53.91 | 122.71 | 41.29 | 36.50 | 22.29 |
| 1083A | 55.48 | 36.46 | 58.76 | 12.60 | 67.62 | 19.01 |
| 1084A | 57.50 | 34.43 | 67.32 | 30.98 | 45.64 | 29.46 |
| 1085A | 81.91 | 56.29 | 89.29 | 36.52 | 45.20 | 33.87 |
| 1086A | 69.11 | 45.07 | 81.61 | 35.83 | 29.92 | 23.79 |
| 1087A | 73.52 | 45.78 | 93.18 | 36.89 | 46.51 | 22.68 |
| 1088A | 78.27 | 47.66 | 97.50 | 28.97 | 60.60 | 13.07 |
| 1089A | 70.77 | 44.59 | 85.21 | 42.99 | 57.18 | 23.69 |
| 1090A | 56.79 | 27.92 | 74.36 | 20.97 | 63.64 | 13.01 |
| 1091A | 57.30 | 33.70 | 73.16 | 49.09 | 54.60 | 12.08 |
| 1092A | 65.72 | 30.80 | 81.07 | 33.63 | 68.71 | 16.65 |
| 1093A | 67.68 | 31.97 | 93.71 | 42.46 | 57.59 | 13.29 |
| 1094A | 54.24 | 26.84 | 63.75 | 34.51 | 59.53 | 11.02 |

**Table S2**

| **Site** | **AQI(N/A)** | **PM_2.5_** | **PM_10_** | **NO_2_** | **O_3_** | **SO_2_** |
| --- | --- | --- | --- | --- | --- | --- |
| 1001A | 97.61 | 63.82 | 104.97 | 38.92 | 119.01 | 3.68 |
| 1002A | 86.41 | 49.87 | 89.49 | 17.37 | 125.03 | 5.80 |
| 1003A | 99.10 | 68.38 | 103.52 | 38.16 | 108.06 | 4.63 |
| 1004A | 97.51 | 65.60 | 97.55 | 39.86 | 112.07 | 7.02 |
| 1005A | 94.26 | 59.11 | 97.30 | 40.02 | 121.50 | 5.04 |
| 1006A | 101.70 | 69.97 | 104.58 | 50.57 | 116.42 | 4.41 |
| 1007A | 97.56 | 64.26 | 110.69 | 38.95 | 112.05 | 3.68 |
| 1008A | 94.06 | 60.92 | 95.10 | 28.14 | 92.15 | 3.79 |
| 1009A | 82.82 | 53.07 | 94.80 | 12.91 | 109.66 | 2.74 |
| 1010A | 84.37 | 52.12 | 86.06 | 24.73 | 110.85 | 2.64 |
| 1011A | 98.57 | 65.42 | 108.49 | 49.30 | 113.06 | 5.63 |
| 1012A | 98.03 | 61.89 | 124.58 | 40.24 | 118.27 | 2.18 |
| **1014A** | **99.57** | **64.35** | **124.72** | **35.10** | **70.70** | **11.65** |
| **1015A** | **97.53** | **65.94** | **122.16** | **32.62** | **78.23** | **8.72** |
| **1016A** | **89.41** | **56.79** | **116.34** | **22.43** | **73.58** | **12.61** |
| **1017A** | **94.13** | **62.12** | **121.89** | **23.20** | **87.82** | **11.29** |
| **1018A** | **104.30** | **71.30** | **124.68** | **33.48** | **72.51** | **15.87** |
| **1019A** | **104.62** | **68.69** | **133.30** | **19.95** | **94.72** | **9.11** |
| **1021A** | **100.46** | **60.07** | **138.11** | **30.41** | **80.47** | **12.95** |
| **1023A** | **112.06** | **81.59** | **125.61** | **31.21** | **71.62** | **7.40** |
| **1024A** | **101.58** | **73.33** | **119.46** | **28.05** | **82.52** | **14.13** |
| **1025A** | **91.59** | **58.14** | **116.05** | **32.59** | **74.16** | **9.37** |
| **1026A** | **91.90** | **61.66** | **116.97** | **16.46** | **84.65** | **10.29** |
| **1027A** | **123.45** | **87.62** | **122.63** | **28.93** | **120.83** | **16.81** |
| 1029A | 129.32 | 86.33 | 186.65 | 27.49 | 46.77 | 13.95 |
| 1030A | 114.67 | 84.40 | 129.84 | 21.05 | 45.81 | 6.03 |
| 1031A | 128.58 | 82.01 | 189.17 | 44.41 | 98.11 | 23.10 |
| 1032A | 107.85 | 76.41 | 134.56 | 18.21 | 31.13 | 11.31 |
| 1033A | 157.11 | 119.37 | 164.34 | 38.35 | 87.03 | 29.68 |
| 1034A | 102.72 | 64.03 | 132.37 | 44.15 | 97.65 | 14.07 |
| 1035A | 130.87 | 94.09 | 157.42 | 13.63 | 143.40 | 40.21 |
| 1036A | 98.87 | 67.29 | 113.76 | 44.22 | 90.55 | 26.21 |
| 1037A | 97.25 | 65.57 | 121.52 | 43.80 | 96.97 | 20.55 |
| 1038A | 101.77 | 69.94 | 127.52 | 41.65 | 100.95 | 40.24 |
| 1039A | 108.02 | 73.32 | 147.69 | 57.59 | 91.80 | 28.50 |
| 1040A | 111.38 | 78.91 | 116.70 | 42.20 | 102.18 | 30.18 |
| 1041A | 107.94 | 76.84 | 120.77 | 43.62 | 96.99 | 42.01 |
| 1042A | 79.88 | 55.02 | 83.03 | 23.79 | 70.83 | 28.81 |
| 1043A | 71.66 | 45.63 | 70.31 | 53.41 | 63.04 | 29.01 |
| 1044A | 81.82 | 46.82 | 106.81 | 42.70 | 49.52 | 22.39 |
| 1045A | 83.05 | 46.89 | 102.52 | 52.25 | 45.79 | 36.07 |
| 1046A | 79.75 | 39.38 | 105.88 | 48.88 | 60.49 | 60.74 |
| 1047A | 109.86 | 65.17 | 161.19 | 49.45 | 28.87 | 26.27 |
| 1048A | 115.44 | 82.31 | 147.63 | 49.94 | 45.03 | 25.73 |
| 1049A | 128.72 | 87.90 | 191.65 | 43.74 | 81.91 | 38.42 |
| 1050A | 119.25 | 87.69 | 138.08 | 18.00 | 71.21 | 35.63 |
| **1051A** | **160.31** | **121.74** | **182.70** | **42.22** | **102.65** | **16.88** |
| **1052A** | **130.88** | **95.74** | **145.01** | **31.47** | **122.93** | **24.41** |
| **1053A** | **121.91** | **87.64** | **162.65** | **35.46** | **65.75** | **14.15** |
| **1054A** | **113.53** | **78.11** | **151.85** | **35.91** | **91.91** | **14.59** |
| **1055A** | **127.26** | **89.27** | **179.09** | **29.96** | **79.34** | **11.07** |
| **1056A** | **132.34** | **91.03** | **166.11** | **36.09** | **92.29** | **22.50** |
| 1057A | 63.51 | 27.91 | 73.90 | 16.51 | 105.91 | 15.30 |
| 1058A | 59.44 | 28.96 | 64.62 | 11.58 | 97.43 | 7.56 |
| 1059A | 59.49 | 21.45 | 71.24 | 21.02 | 98.04 | 18.68 |
| 1060A | 74.00 | 37.65 | 92.03 | 21.25 | 97.32 | 13.56 |
| 1061A | 81.46 | 39.13 | 102.87 | 19.89 | 104.02 | 12.80 |
| 1062A | 72.20 | 35.12 | 86.85 | 41.83 | 84.83 | 9.69 |
| 1063A | 67.66 | 31.84 | 73.04 | 26.20 | 81.77 | 4.76 |
| 1064A | 71.47 | 39.98 | 75.15 | 30.33 | 100.44 | 11.80 |
| 1065A | 67.75 | 28.23 | 75.92 | 25.22 | 98.74 | 14.44 |
| 1066A | 62.94 | 29.65 | 68.04 | 16.10 | 74.20 | 6.19 |
| **1067A** | **108.30** | **73.76** | **142.29** | **43.72** | **94.51** | **12.88** |
| **1068A** | **113.64** | **79.41** | **165.11** | **45.31** | **99.95** | **7.52** |
| **1069A** | **111.04** | **78.95** | **136.77** | **34.66** | **106.60** | **8.99** |
| **1070A** | **93.54** | **65.50** | **106.52** | **47.53** | **114.86** | **10.43** |
| 1071A | 98.56 | 70.85 | 101.90 | 40.93 | 98.39 | 27.50 |
| 1072A | 110.03 | 79.45 | 108.83 | 31.98 | 122.68 | 22.10 |
| 1073A | 88.94 | 62.04 | 91.09 | 35.50 | 108.23 | 19.59 |
| 1074A | 125.74 | 93.46 | 134.83 | 25.54 | 40.07 | 12.60 |
| 1075A | 113.90 | 83.99 | 111.35 | 32.01 | 71.78 | 18.63 |
| 1076A | 131.77 | 98.88 | 132.61 | 33.54 | 99.14 | 20.61 |
| 1077A | 128.02 | 95.48 | 126.60 | 49.28 | 77.30 | 24.40 |
| 1078A | 125.94 | 92.21 | 142.29 | 57.12 | 70.82 | 39.31 |
| 1079A | 113.91 | 83.92 | 133.87 | 49.47 | 58.73 | 24.22 |
| 1080A | 104.83 | 74.26 | 125.28 | 49.23 | 83.04 | 29.30 |
| 1081A | 83.64 | 51.65 | 115.61 | 31.97 | 33.09 | 19.64 |
| 1082A | 90.99 | 52.54 | 128.10 | 36.35 | 31.05 | 20.48 |
| 1083A | 59.20 | 38.49 | 66.69 | 14.43 | 34.62 | 19.29 |
| 1084A | 71.89 | 46.32 | 91.94 | 25.47 | 33.32 | 27.37 |
| 1085A | 83.44 | 56.97 | 102.87 | 28.39 | 33.11 | 28.12 |
| 1086A | 70.95 | 44.06 | 90.10 | 31.52 | 18.61 | 31.92 |
| 1087A | 79.92 | 49.11 | 103.25 | 30.18 | 36.37 | 27.36 |
| 1088A | 86.28 | 50.90 | 117.34 | 33.28 | 81.32 | 19.10 |
| 1089A | 85.26 | 51.39 | 109.87 | 47.32 | 79.29 | 23.09 |
| 1090A | 73.32 | 25.40 | 100.20 | 21.76 | 84.96 | 15.21 |
| 1091A | 64.94 | 27.55 | 80.06 | 42.47 | 78.05 | 14.41 |
| 1092A | 81.26 | 22.31 | 92.50 | 32.83 | 110.54 | 15.64 |
| 1093A | 82.12 | 27.40 | 111.68 | 42.13 | 81.53 | 17.59 |
| 1094A | 68.32 | 22.96 | 79.59 | 32.28 | 96.57 | 9.98 |
| 1095A | 68.78 | 22.07 | 85.41 | 36.06 | 90.59 | 9.19 |
| 1096A | 82.32 | 27.80 | 109.97 | 39.27 | 87.85 | 15.26 |
| 1097A | 69.21 | 19.39 | 85.83 | 21.07 | 82.19 | 7.97 |
| 1098A | 55.15 | 33.84 | 47.87 | 16.37 | 90.05 | 9.72 |
| 1099A | 57.32 | 26.02 | 59.06 | 34.55 | 88.20 | 13.59 |
| 1100A | 63.45 | 36.90 | 67.69 | 29.81 | 86.50 | 17.26 |

**Table S3**

| **Time period** | **Southern Air mass** | | **Northern Air mass** | |
| --- | --- | --- | --- | --- |
|  | 2015/8/5 | 28.81 |  |  |
|  | 2015/8/6 | 20.14 |  |  |
|  | 2015/8/7 | 20.08 | 2015/8/8 | 22.25 |
|  | 2015/8/12 | 22.27 | 2015/8/9 | 20.26 |
| pre-Parade | 2015/8/13 | 23.29 | 2015/8/10 | 15.61 |
|  | 2015/8/16 | 20.12 | 2015/8/11 | 16.88 |
|  | 2015/8/17 | 19.82 | 2015/8/14 | 11.55 |
|  | 2015/8/18 | 46.22 | 2015/8/15 | 13.37 |
|  | 2015/8/19 | 30.33 |  |  |
| **Mean`** | - | **25.68** | - | **16.65** |
|  |  |  | 2015/8/20 | 21.52 |
|  |  |  | 2015/8/21 | 19.98 |
|  |  |  | 2015/8/22 | 10.55 |
|  |  |  | 2015/8/23 | 14.97 |
|  | 2015/8/28 | 11.21 | 2015/8/24 | 11.06 |
| Parade | 2015/8/30 | 14.51 | 2015/8/25 | 9.72 |
|  | 2015/9/3 | 9.675 | 2015/8/26 | 10.98 |
|  |  |  | 2015/8/27 | 11.92 |
|  |  |  | 2015/8/31 | 12.09 |
|  |  |  | 2015/9/1 | 7.67 |
|  |  |  | 2015/9/2 | 9.14 |
| **Mean** |  | **11.80** |  | **12.69** |
|  | 2015/9/4 | 25.36 |  |  |
|  | 2015/9/7 | 32.05 |  |  |
|  | 2015/9/8 | 24.62 | 2015/9/5 | 19.32 |
|  | 2015/9/13 | 22.90 | 2015/9/6 | 13.80 |
|  | 2015/9/14 | 47.52 | 2015/9/9 | 12.36 |
| post-Parade | 2015/9/15 | 25.67 | 2015/9/10 | 10.71 |
|  | 2015/9/16 | 37.34 | 2015/9/12 | 12.17 |
|  | 2015/9/17 | 26.94 | 2015/9/18 | 16.65 |
|  | 2015/9/20 | 32.15 | 2015/9/19 | 19.29 |
|  | 2015/9/21 | 46.19 |  |  |
| **Mean** |  | **32.07** |  | **14.90** |

**Supplementary Figures**

**Figure S1. A map of Beijing and its surrounding cities.** The red five-pointed star indicates the MAX-DOAS observation site at Beijing urban area (40°N, 116°22′48″E), and the blue circles represent the other surrounding regions would be mentioned in this paper. The map was generated by Adobe Illustrator CS6 (<http://www.adobe.com/cn/products/illustrator.html>). Base map is from Google Earth 7.1.5.1557 (<http://www.google.cn/intl/zh-CN/earth/>).

**Figure S2. For the “Cycle 1” (From August 14^th^ to 21^st^) cluster analysis of the 24-**h AMBTs starting at 500m in Beijing urban area. There could be retrieved an air mass back trajectory in every hour, which means it could be produced 24 AMBTs during a full day. Cluster analysis could sort these AMBTs by directions and the color lines denote the 5 categories directions of AMBTs. The “Peak” means averaged tropospheric NO_2_ VCDs at the culminating point in the entire cycle, and the “Valley” signifies the mean tropospheric NO_2_ VCDs in the bottom position of the cycle. The rest was transition value in the cycle. Base map is from TrajStat 1.2.2 software (http://www.meteothinker.com).

**Figure S3. For the “Cycle 2” (From September 6^th^ to 9^th^) cluster analysis of the 24-h AMBTs starting at 500m in Beijing urban area.** Base map is from TrajStat 1.2.2 software (http://www.meteothinker.com).

**Figure S4. For the “Cycle 3” (From September 12^rd^ to 18^st^) cluster analysis of the 24-h AMBTs starting at 500m in Beijing urban area.** Base map is from TrajStat 1.2.2 software (http://www.meteothinker.com).

**Figure S5. Cluster analysis of the 24-h AMBTs for southern and northern air mass on Nov. 4 and 7 (two high value days) starting at 500m in Beijing urban region.** The black lines mean the AMBTs during the whole day for every hour (which have been signed in title). And the color lines present the 6 categories directions of AMBTs. Base map is from TrajStat 1.2.2 software (http://www.meteothinker.com).

**Figure S6. The PSCF maps for NO_2_ VCDs for the periods of pre-Parade, Parade, post-Parade and pre-Parade + post-Parade, respectively.** Base map is from TrajStat 1.2.2 software (http://www.meteothinker.com).

**Figure S7. The PSCF maps for NO_2_ VCDs for the periods of pre-APEC, APEC, post-APEC and pre-APEC+ post-APEC, respectively.** Base map is from TrajStat 1.2.2 software (http://www.meteothinker.com).

**Figure S8. Model domain and topographic field (m).** The map was generated using NCAR Command Language (NCL) Version 6.2 (https://www.earthsystemgrid.org/dataset/ncl.620.html).

**Figure S9. Comparison between radiosonde-observed water vapor mixing ratio in Beijing (black open circles) and corresponding simulations (red lines) at 00:00 UTC during 1-20 November, 2014.**

**Figure S10. Comparisons between radiosonde-observed water vapor mixing ratio in Beijing (black open circles) and corresponding simulations (red lines) at 00:00 UTC from 22 August to 10 September, 2015.**


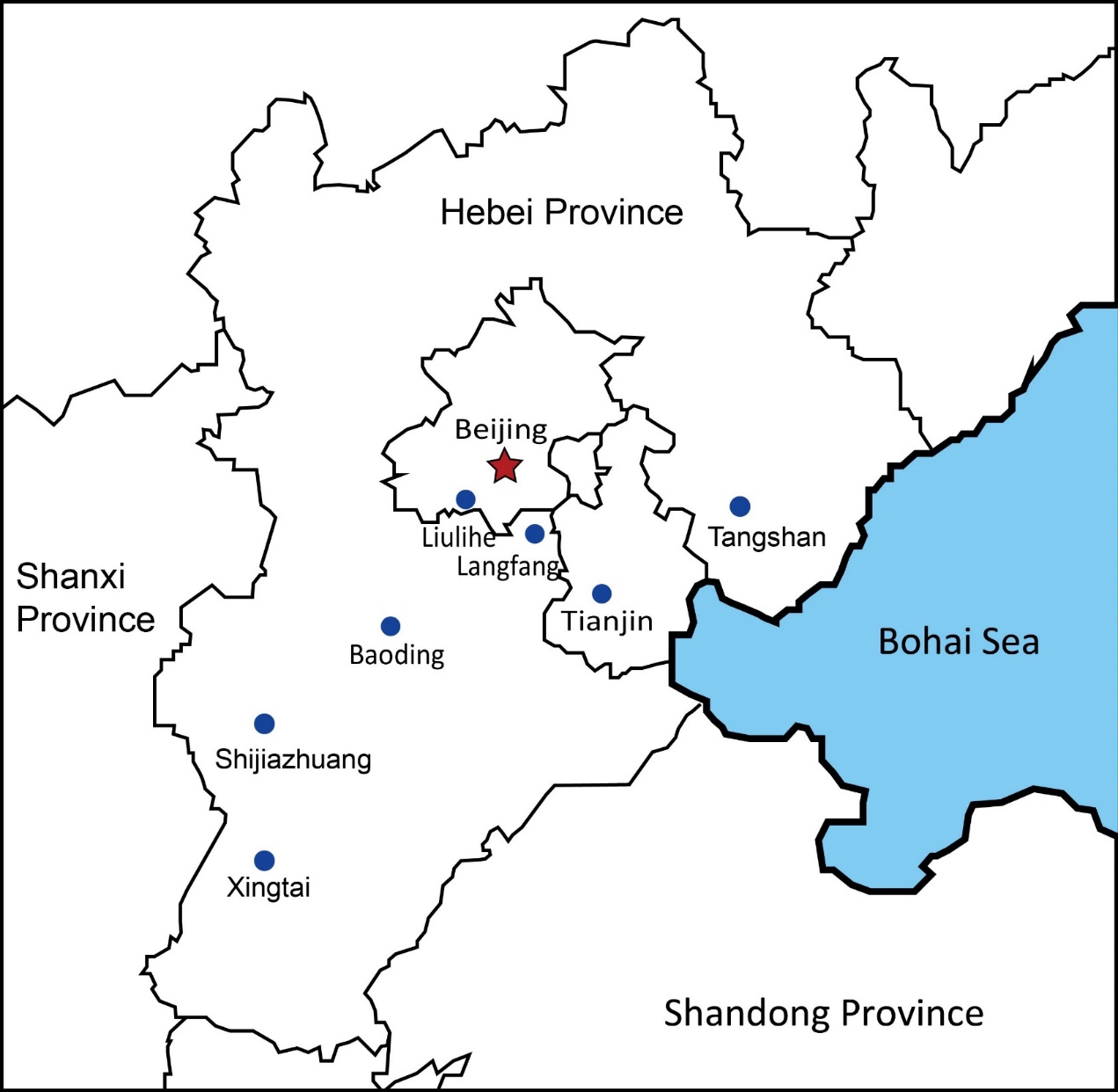


**Fig.S1**


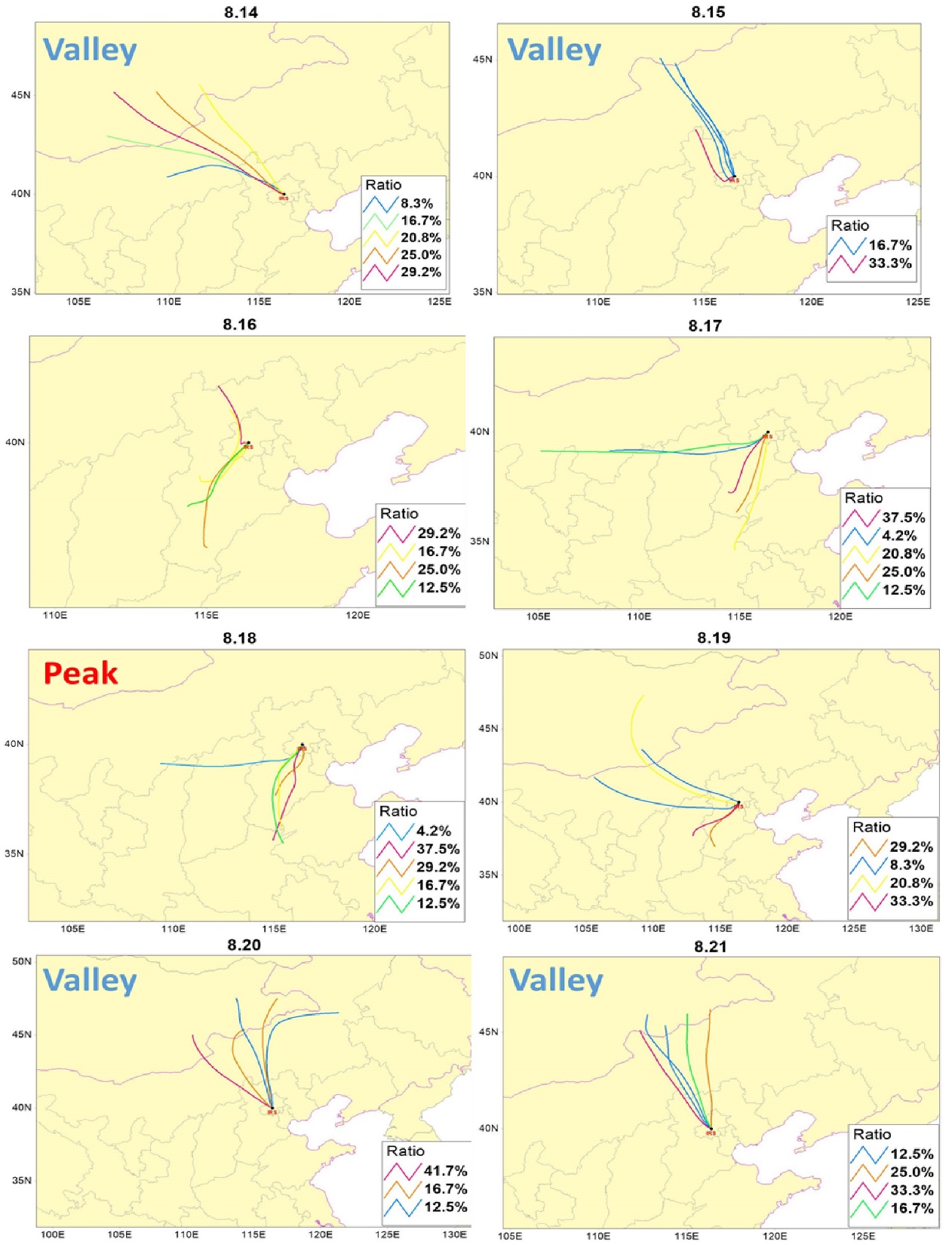


**Fig.S2**


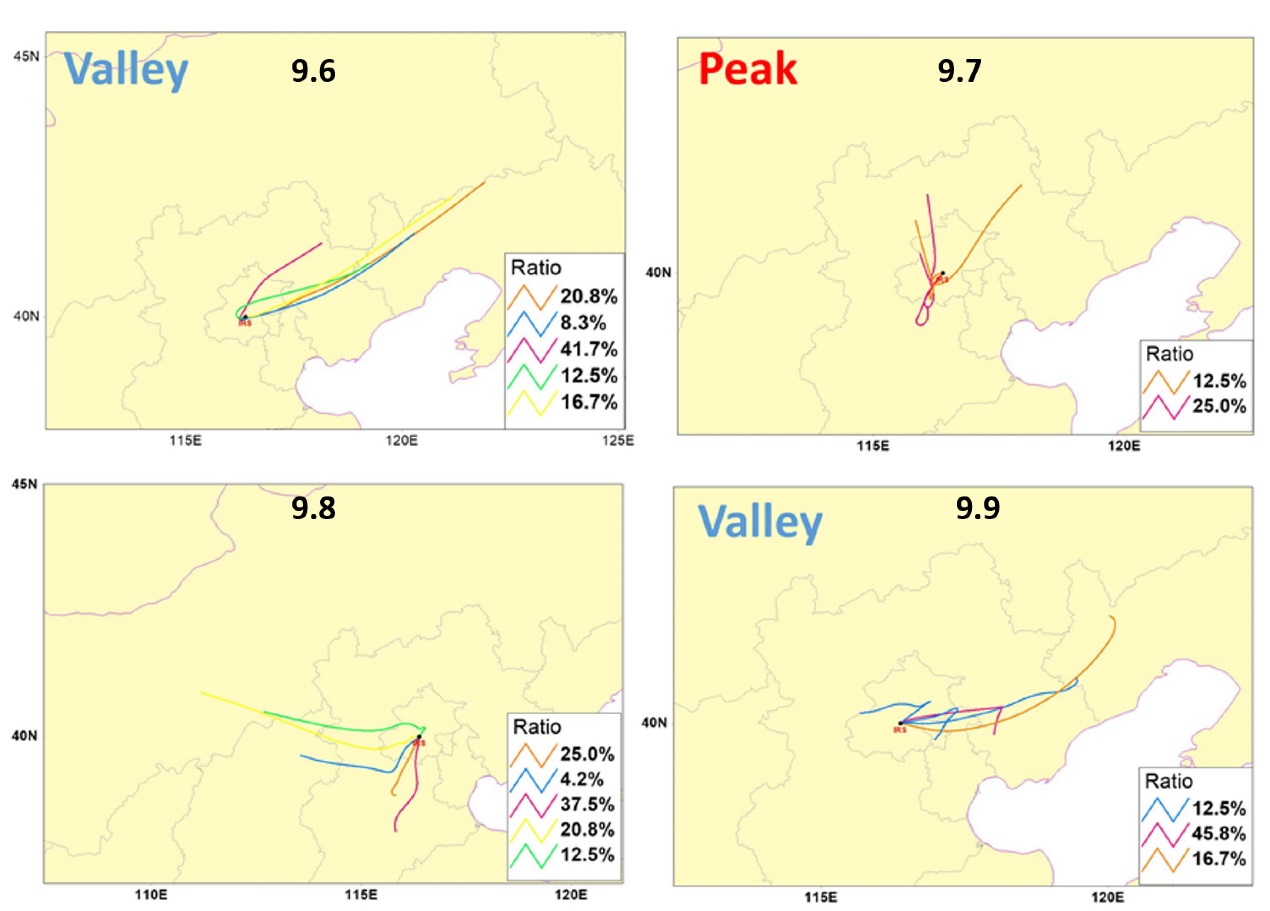


**Fig.S3**


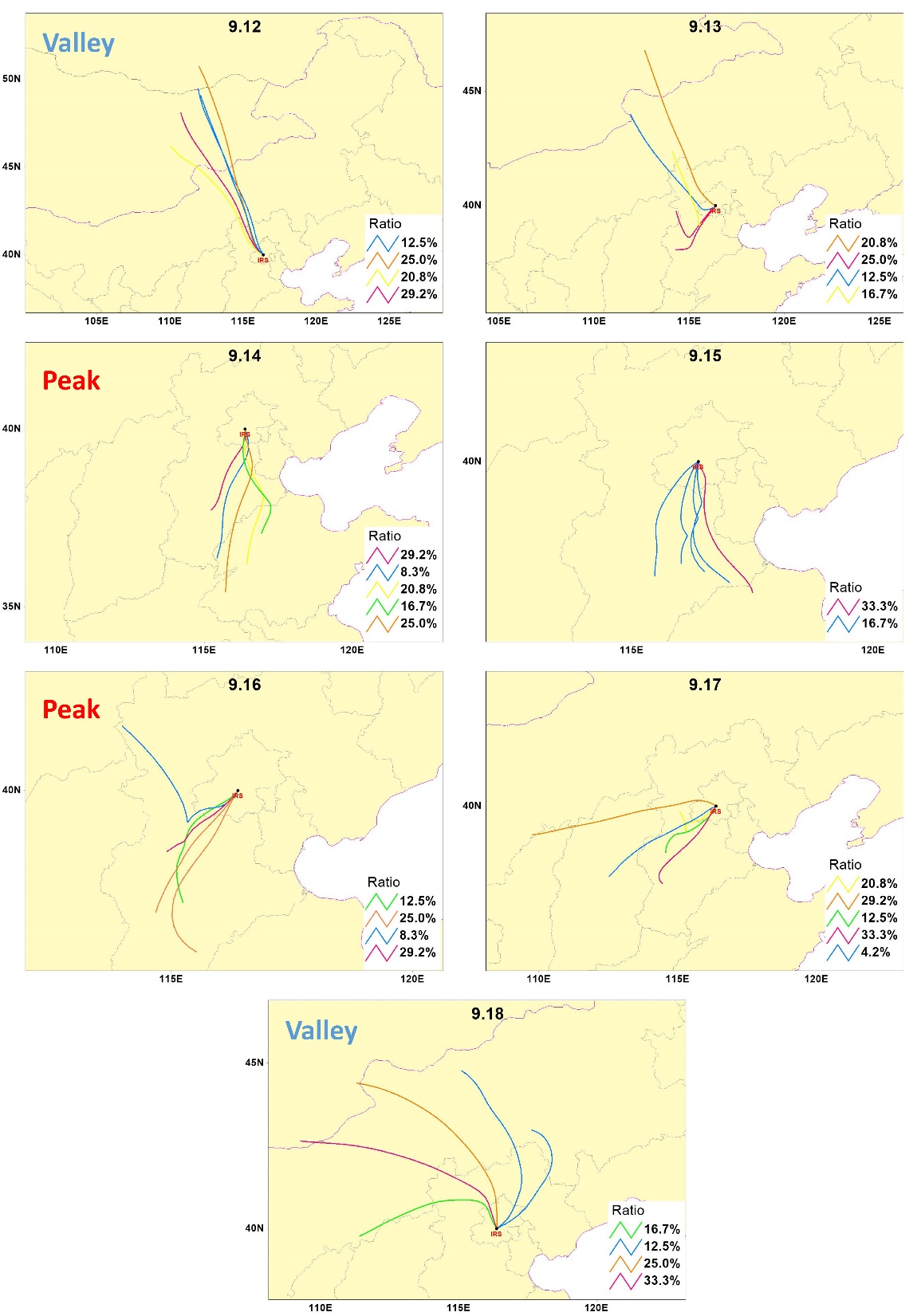


**Fig.S4**


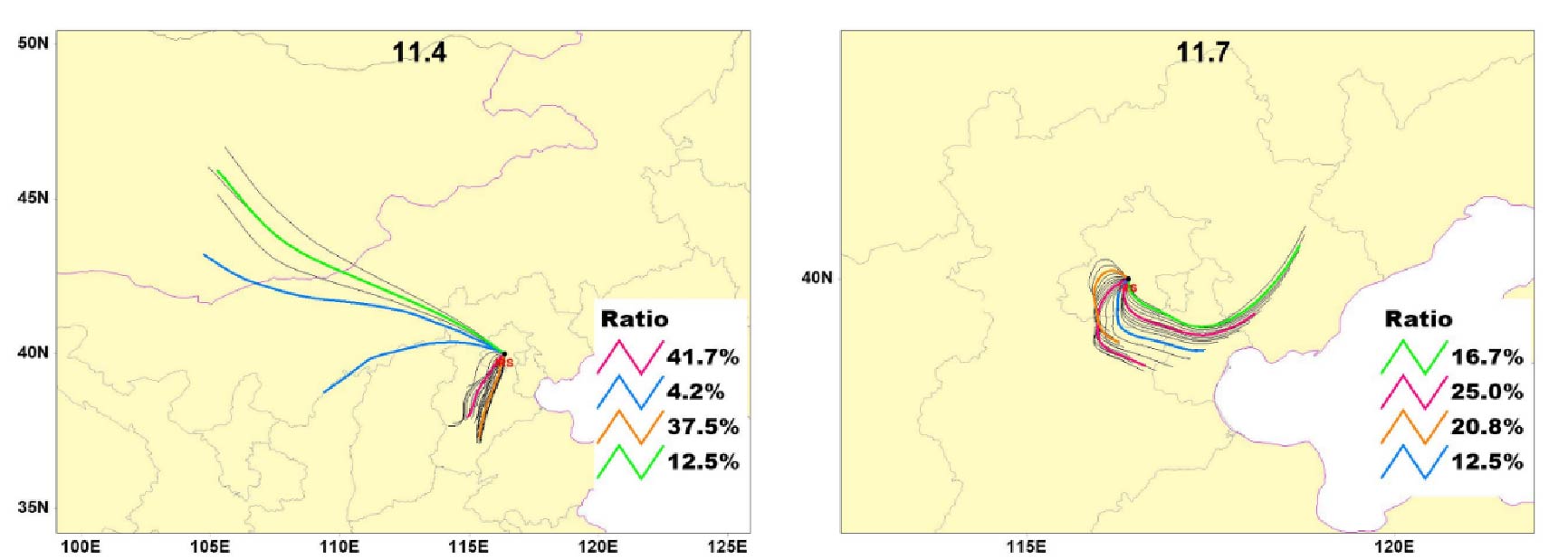


**Fig.S5**

**
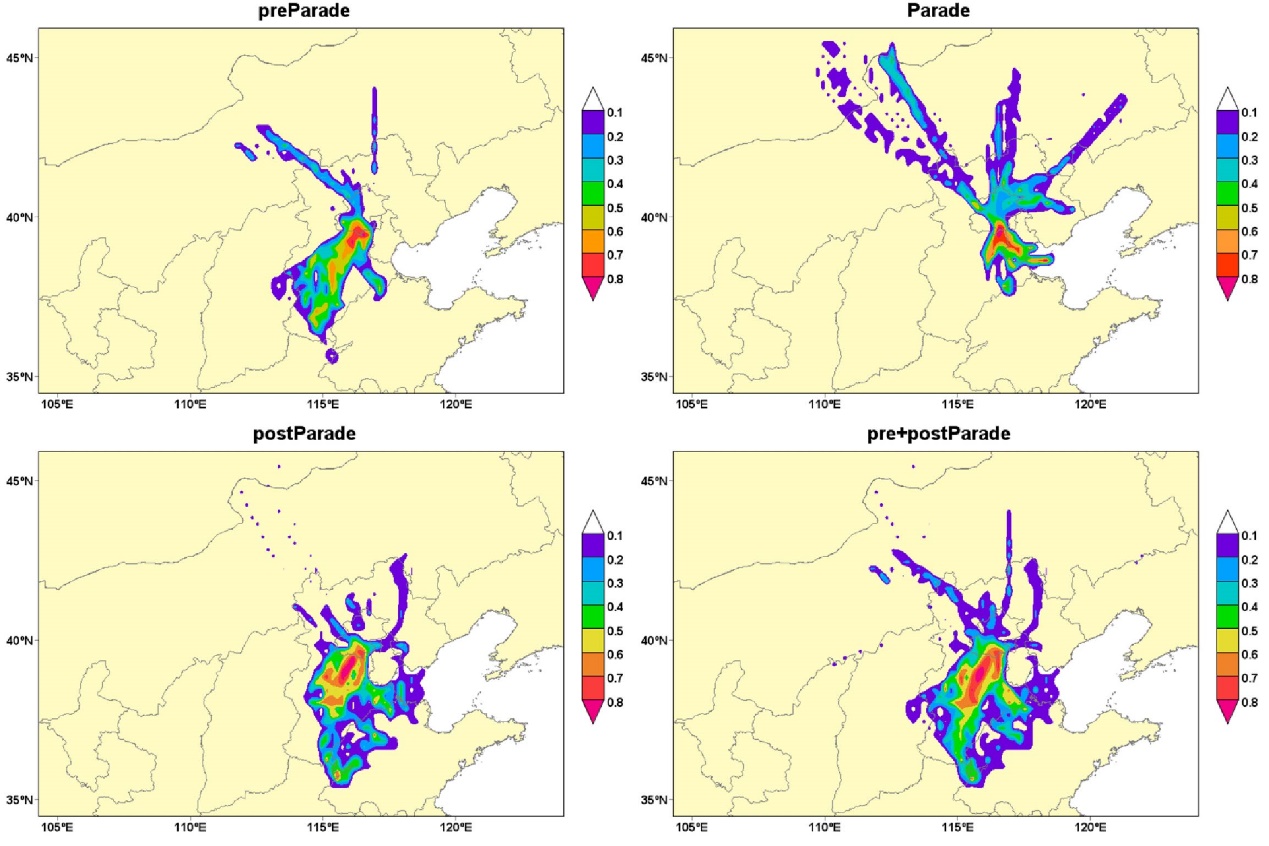
Fig.S6**


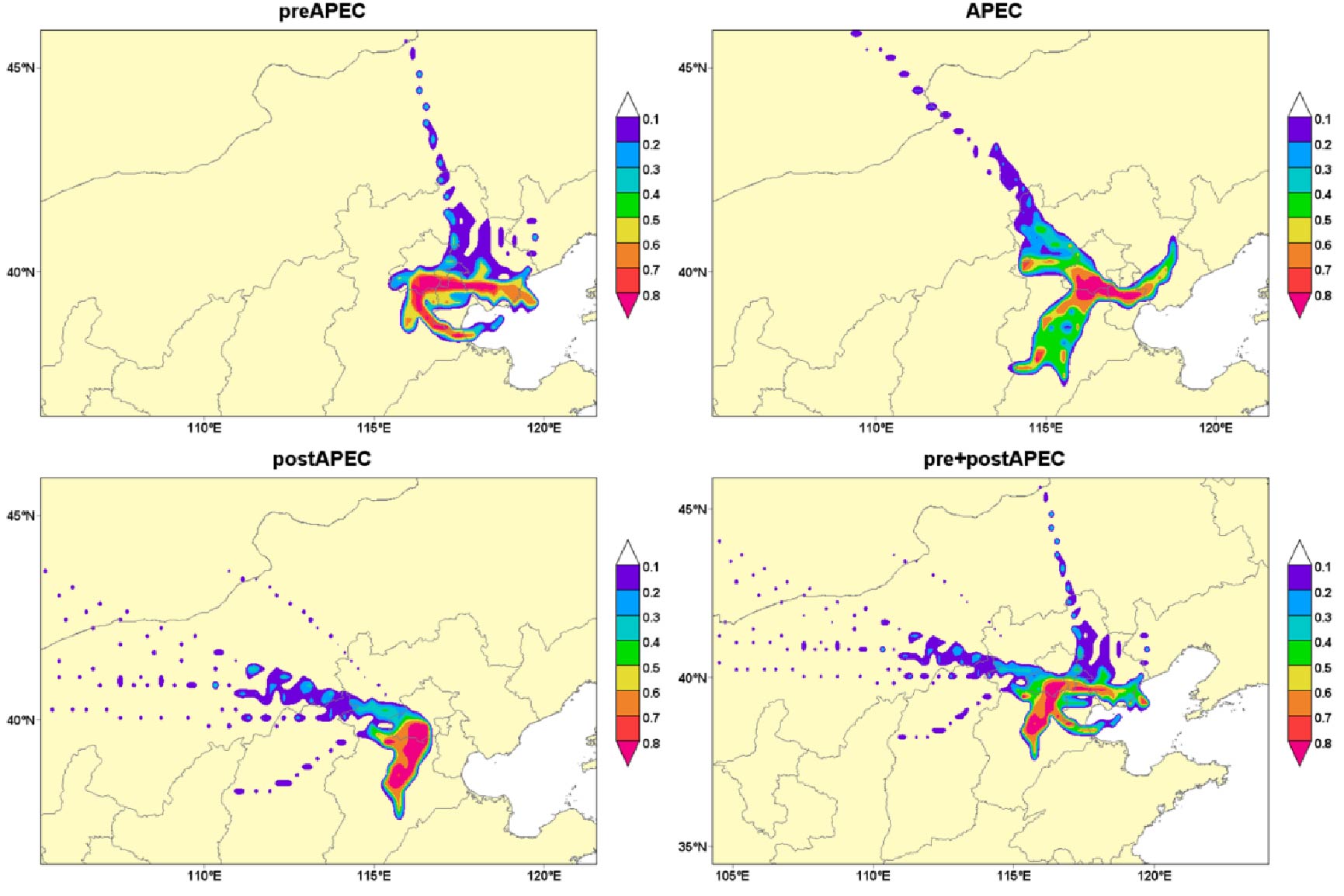


**Fig.S7**


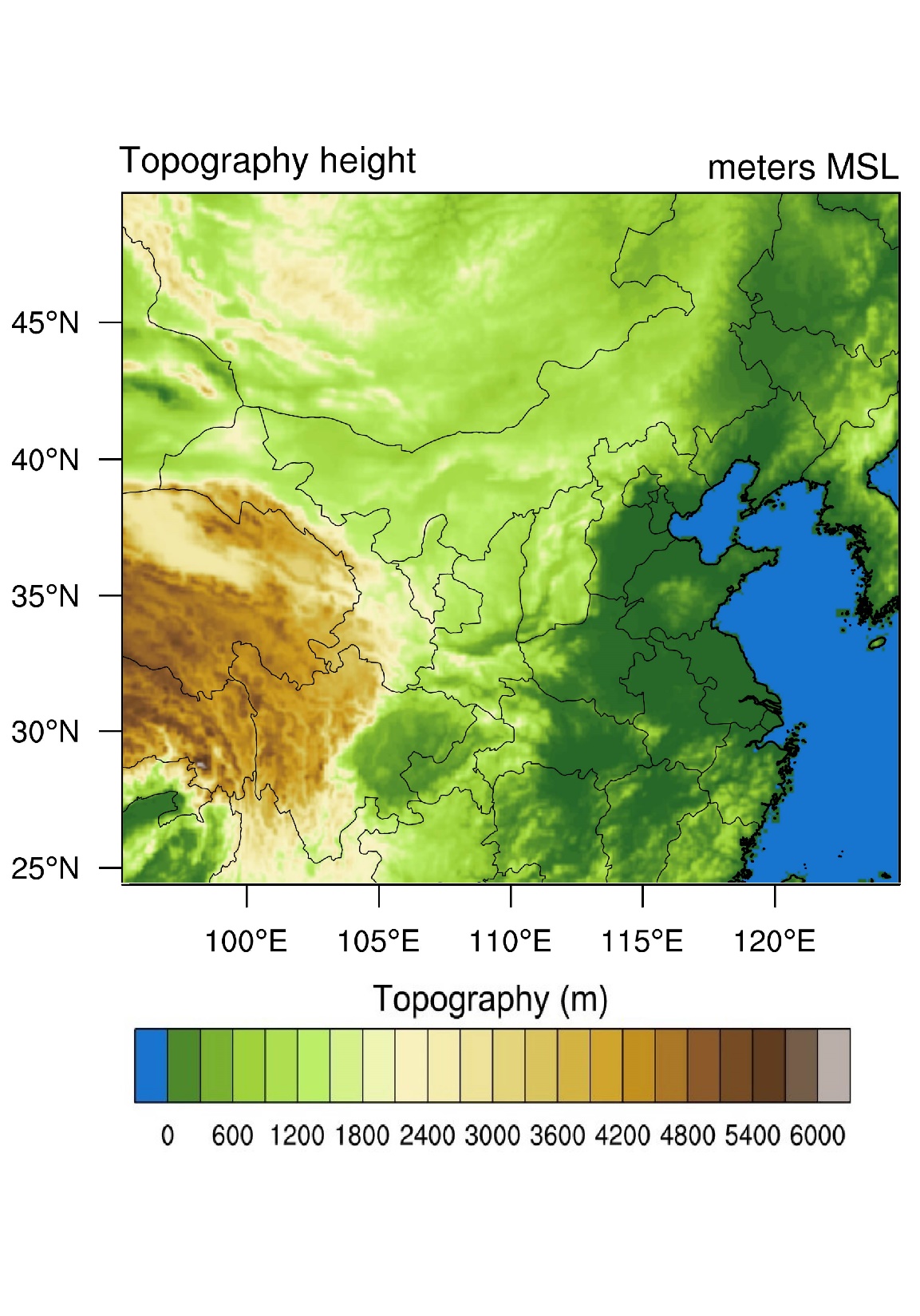


**Fig.S8**


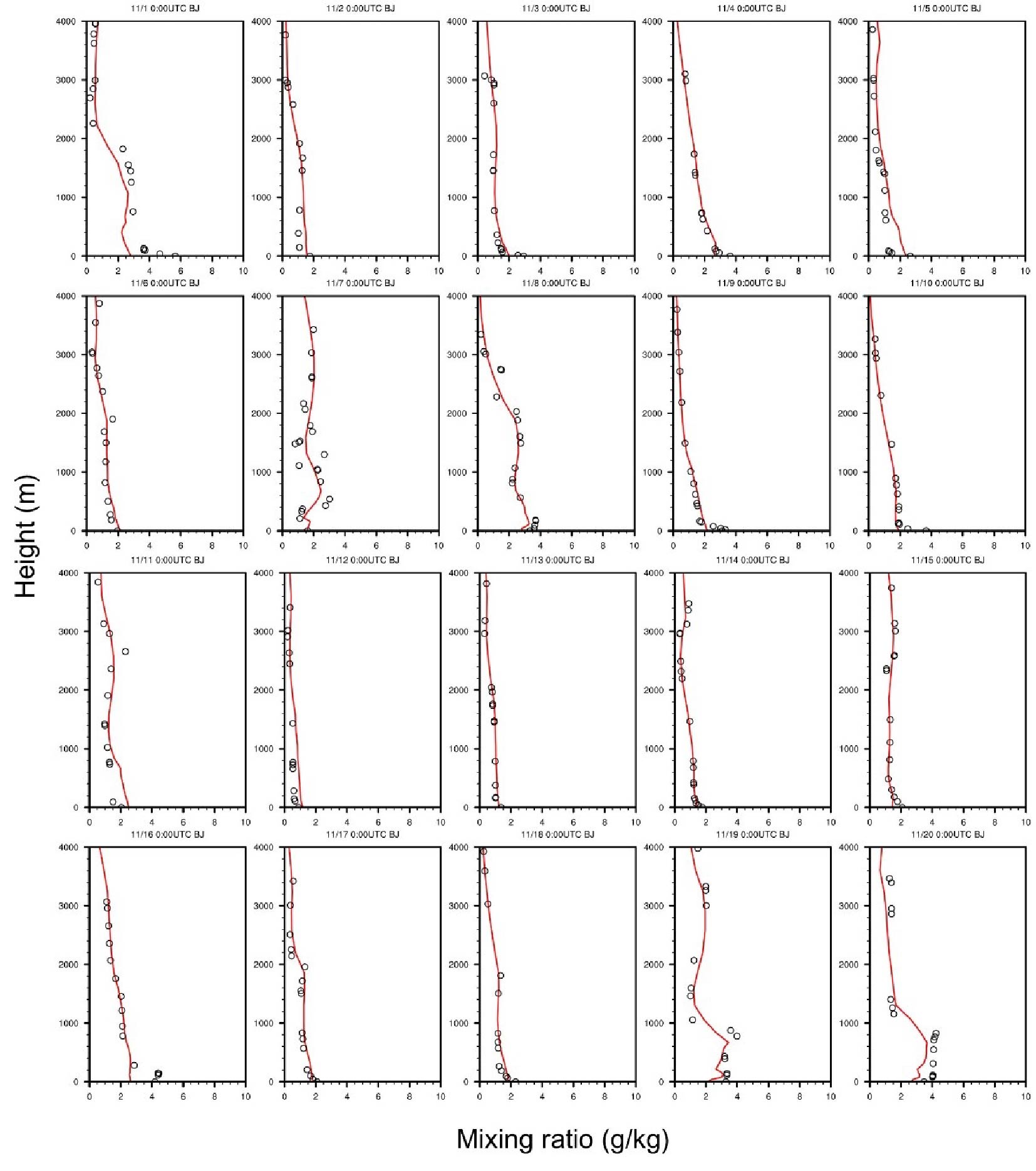


**Fig.S9**


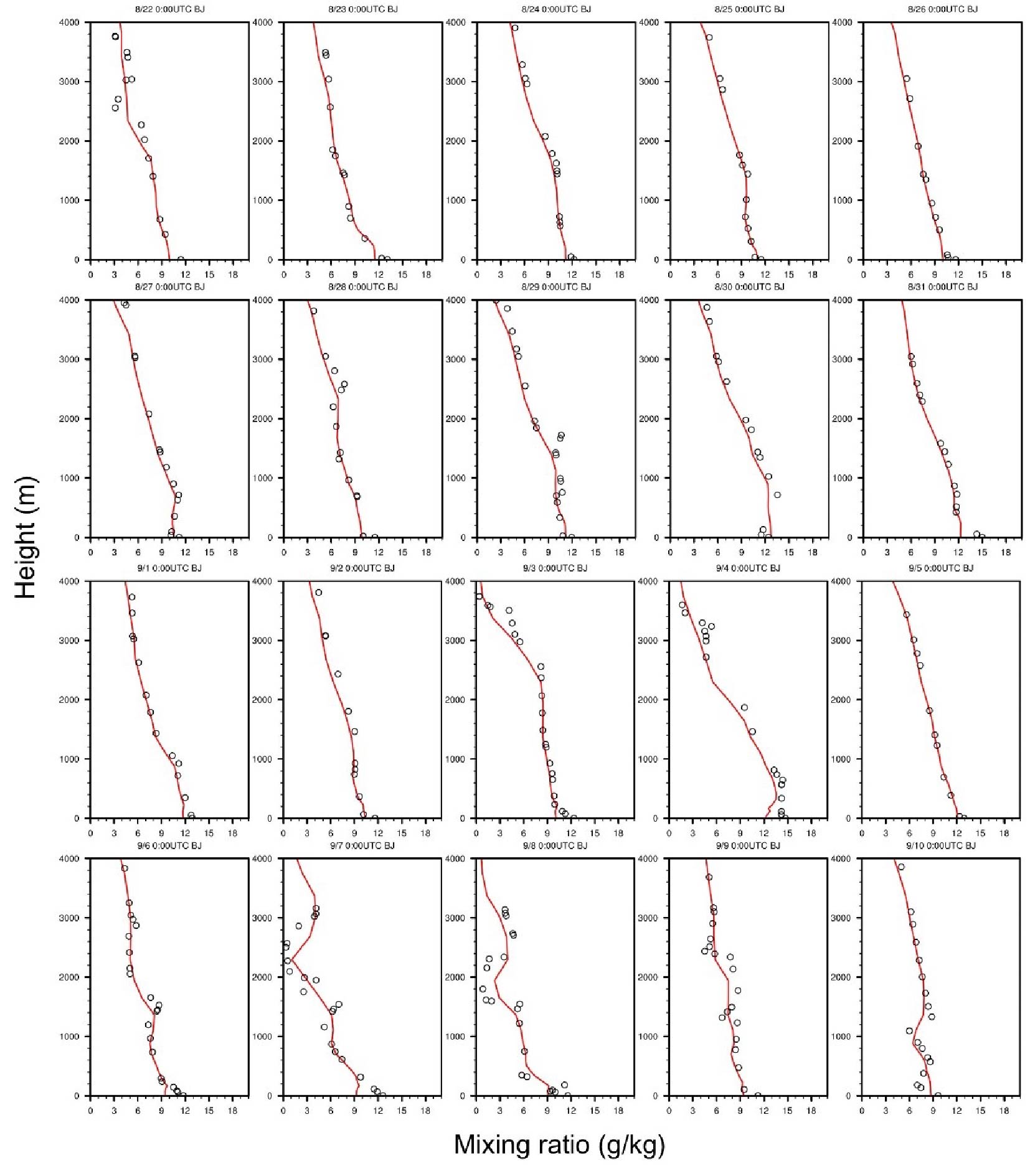


**Fig.S10**
